# Data-Driven Modeling of Linear Dynamical Systems with Quadratic Output in the AAA Framework

**DOI:** 10.1007/s10915-022-01771-5

**Published:** 2022-02-28

**Authors:** Ion Victor Gosea, Serkan Gugercin

**Affiliations:** 1 Data-Driven System Reduction and Identification (DRI) Group, Max Planck Institute for Dynamics of Complex Technical Systems, Sandtorstrasse 1, 39106 Magdeburg, Germany; 2 Department of Mathematics and Computational Modeling and Data Analytics Division, Academy of Data Science, Virginia Tech, Blacksburg, VA 24061 USA

**Keywords:** Data-driven modeling, Model reduction, Nonlinear dynamics, Interpolation, Least-squares fit, Barycentric form

## Abstract

We extend the Adaptive Antoulas-Anderson (AAA) algorithm to develop a data-driven modeling framework for linear systems with quadratic output (LQO). Such systems are characterized by two transfer functions: one corresponding to the linear part of the output and another one to the quadratic part. We first establish the joint barycentric representations and the interpolation theory for the two transfer functions of LQO systems. This analysis leads to the proposed AAA-LQO algorithm. We show that by interpolating the transfer function values on a subset of samples together with imposing a least-squares minimization on the rest, we construct reliable data-driven LQO models. Two numerical test cases illustrate the efficiency of the proposed method.

## Introduction

Model order reduction (MOR) is used to approximate large-scale dynamical systems with smaller ones that ideally have similar response characteristics to the original. This has been an active research area and many approaches to MOR have been proposed. We refer the reader to [1, 3, 6, 9, 37, 39] and the references therein for an overview of MOR methods for both linear and nonlinear dynamical systems.

MOR, as the name implies, assumes access to a full order model to be reduced; in most cases, in the form of a state-space formulation obtained via, e.g., a spatial discretization of the underlying partial differential equations. Then, the reduced order quantities are computed via an explicit projection of the full-order quantities. However, in some cases, access to the original (full order) dynamics is not available. Instead, one has access to an abundant amount of data, such as input/output measurements, snapshots of the state variable in the time domain, or evaluations of the transfer function(s) in the frequency domain. In this case, the goal is to construct an approximant (surrogate model) directly from data, which we refer to as data-driven modeling. This is the framework we consider in this paper. Such scenarios arise frequently in many applications such as circuit modeling where the modeling of distributed/integrated circuits characterized by many components is done by the frequency-domain data using, e.g., the S-parameters [27]. Structural dynamics is another example. Even when a mathematical model of a highly complex physical structure is not available, the structural (displacement and velocity) time and frequency domain responses can be measured accurately at specific locations on the structures, thanks to the advances in testing capabilities and the near ubiquitous deployment of high bandwidth sensing [30]. We refer the reader to [3, 5, 8, 12, 16, 26, 36] and the references therein for more details on data-driven modeling.

Specifically, we focus on data-driven modeling of linear dynamical systems with quadratic output (LQO). In our formulation, data correspond to frequency domain samples of the input/output mapping of the underlying LQO system, in the form of samples of its two transfer functions: the first transfer function being a single-variable one and the second a bivariate one. For this data set, the proposed framework first develops the barycentric rational interpolation theory for LQO systems to interpolate a subset of the data and then extends the AAA algorithm [32] to this setting by minimizing a least-square measure in the remaining data.

We note that system identification of general nonlinear systems has been a popular topic. In particular, we mention here the special case of identifying linear systems with nonlinear output or input functions, e.g., the so-called Wiener [43] and Hammerstein models, respectively. Significant effort has been allocated for identification of such models; see, for example, [17, 23], and the references therein. Nevertheless, the methods previously mentioned are based in the time domain, while in this paper we focus on frequency domain data. We point out that the frequency-data based Loewner framework was recently extended to identifying Hammerstein models in [24].

The rest of the paper is organized as follows: We discuss LQO systems and their transfer functions in Sect. 2, followed by a review of barycentric rational approximation for linear systems and the AAA algorithm in Sect. 3. Next, we develop the theory for barycentric representation and multivariate interpolation for LQO systems in Sect. 4. Based on this analysis, in Sect. 5, we present the proposed algorithm, AAA-LQO, for data-driven modeling of LQO systems. The numerical experiments are given in Sect. 6 followed by the conclusions in Sect. 7.

## Linear Systems with Quadratic Output

In state-space form, linear dynamical systems with quadratic output (LQO systems) are described as

2.1 $$\begin{aligned} \Sigma _{\textsf {LQO}}: {\left\{ \begin{array}{ll} {\dot{\mathbf{x}}}(t)=\mathbf{A}\mathbf{x}(t)+\mathbf{b} u(t),\\  y(t)=\mathbf{c}^T\mathbf{x}(t) +{\mathbf{K}\big [ \mathbf{x}(t) \otimes \mathbf{x}(t) \big ]}, \end{array}\right. } \end{aligned}$$

where $\mathbf{A}\in {\mathbb {R}}^{ {{\mathcal {N}}} \times {{\mathcal {N}}} }$, $\mathbf{b}, \mathbf{c}\in {\mathbb {R}}^{ {{\mathcal {N}}} }$, $\mathbf{K}\in {\mathbb {R}}^{1 \times {{\mathcal {N}}} ^2}$, and the symbol $\otimes$ denotes the Kronecker product, i.e., for the vector $\mathbf{x}= [x_1 \ x_2 \ \cdots \ x_ {{\mathcal {N}}} ]^T \in {\mathbb {R}}^{ {{\mathcal {N}}} }$, we have

$$\begin{aligned} \mathbf{x}\otimes \mathbf{x}= [x_1^2 \ \ x_1 x_2 \ \ x_1 x_3 \ \ \cdots \ \ x_1 x_ {{\mathcal {N}}} \ \ \cdots x_ {{\mathcal {N}}} ^2]^T \in {\mathbb {R}}^{ {{\mathcal {N}}} ^2}. \end{aligned}$$

In (2.1), $\mathbf{x}(t) \in {\mathbb {R}}^{ {{\mathcal {N}}} }, u(t) \in {\mathbb {R}}$, and $y(t) \in {\mathbb {R}}$, are, respectively, the states, input, and output of $\Sigma _{\textsf {LQO}}$. The quadratic part of the output in (2.1), $\mathbf{K}\big [ \mathbf{x}(t) \otimes \mathbf{x}(t) \big ]$, can be rewritten as $\mathbf{x}^T(t) \mathbf{M}\mathbf{x}(t)$ with $\mathbf{M}\in {\mathbb {R}}^{ {{\mathcal {N}}} \times {{\mathcal {N}}} }$ with $\mathbf{K}= \mathsf {vec}(\mathbf{M}))$ where $\mathsf {vec}(\cdot )$ denotes the vectorization operation. In some cases, $\mathbf{c}= \mathbf {0}$ in (2.1), and thus the output has only the quadratic term.

The class of dynamical systems (2.1) considered in this paper is particularly useful when the observed quantity of interest is given by the variance or deviation of the state variables from a reference point [7]. Particular examples are random vibrations analysis [29] and applications in which the observed output is expressed as an energy or power quantity [7].

Several projection-based MOR methodologies have been already proposed for LQO systems. More precisely, balanced truncation-type methods were considered in [7, 35, 41], while interpolation-based methods were used in [19, 42]. All these methods explicitly work with the state-space matrices $\mathbf{A},\mathbf{b},\mathbf{c}$ and $\mathbf{K}$ in (2.1). The main goal of this work is to develop a *data-driven* modeling framework for LQO systems where only input-output measurements, in the form of transfer function evaluations, are needed as opposed to a state-space representation. Therefore, we first discuss the transfer functions of this special class of dynamical systems.

### Transfer Functions of LQO Systems

Many classes of nonlinear systems can be represented in the time domain by generalized kernels as presented in the classical Wiener or Volterra series representations. Generically, infinite number of kernels appear in such series, corresponding to each homogeneous subsystem. For more details we refer the reader to [38, 43].

For the LQO system (2.1), the nonlinearity is present in the state-to-output equation only and one can write the input-output mapping of the system in the frequency domain using two transfer functions: (i) one corresponding to the linear part of the output, i.e., $y_1(t) = \mathbf{c}^T \mathbf{x}(t)$ and (ii) one corresponding to the quadratic part of the output, i.e., $y_2(t) = \mathbf{K}(\mathbf{x}(t) \otimes \mathbf{x}(t))$. These transfer functions were recently derived in [19] using their time-domain representations. In the next result, we introduce and re-derive them for the completeness of the paper and to illustrate to the reader how they naturally appear.

#### Lemma 2.1

Consider the LQO system in (2.1) with $\mathbf{x}(0) = \mathbf {0}$. Let the input *u*(*t*) be a sum of the *J* harmonic terms, i.e.,

2.2 $$\begin{aligned} u(t) = \sum _{j =1}^J e^{\mathrm{i}\omega _j t },~~~~\text{ where }~~\omega _j > 0~~\text{ for }~~j=1,2,\ldots ,J,~~ \end{aligned}$$

and $\mathrm{i}^2 = -1$. Then, the output in steady-state is given by

2.3 $$\begin{aligned} y_{\mathrm{ss}}(t) = \sum _{j=1}^J H_1(\mathrm{i}\omega _j) e^{\mathrm{i}\omega _j t} + \sum _{j=1}^J \sum _{\ell =1}^J H_2(\mathrm{i}\omega _j,\mathrm{i}\omega _\ell ) e^{\mathrm{i}(\omega _j + \omega _\ell )t}, \end{aligned}$$

where

2.4 $$\begin{aligned} H_1(s) = \mathbf{c}^T(s\mathbf{I}_ {{\mathcal {N}}} -\mathbf{A})^{-1} \mathbf{b}\end{aligned}$$

is the single-variable rational transfer function corresponding to $y_1(t)$ and

2.5 $$\begin{aligned} H_2(s,z) = \mathbf{K}\Big [ (s\mathbf{I}_ {{\mathcal {N}}} -\mathbf{A})^{-1} \mathbf{b}\otimes (z\mathbf{I}_ {{\mathcal {N}}} -\mathbf{A})^{-1} \mathbf{b}\Big ] \end{aligned}$$

is the two-variable rational transfer function corresponding to $y_2(t)$ with $\mathbf{I}_ {{\mathcal {N}}}$ denoting the identity matrix of size ${{\mathcal {N}}} \times {{\mathcal {N}}}$.

#### Proof

For the input *u*(*t*) in (2.2) and with $\mathbf{x}(0) = \mathbf {0}$, the solution of the linear state-equation in (2.1) in steady-state can be written as a sum of scaled complex exponential functions as

2.6 $$\begin{aligned} \mathbf{x}_{\mathrm{ss}}(t) = \sum _{j=1}^J \mathbf{G}_1(\mathrm{i}\omega _j) e^{\mathrm{i}\omega _j t}, \end{aligned}$$

where $\mathbf{G}_1(s) = (s\mathbf{I}_ {{\mathcal {N}}} -\mathbf{A})^{-1} \mathbf{b}$. Substituting (2.6) into the output equation of (2.1), we obtain

2.7 $$\begin{aligned} \begin{aligned} y_{\mathrm{ss}}(t)&= \mathbf{c}^T \sum _{j=1}^J \mathbf{G}_1(\mathrm{i}\omega _j) e^{\mathrm{i}\omega _j t} + \mathbf{K}\big [ \sum _{j=1}^J \mathbf{G}_1(\mathrm{i}\omega _j) e^{\mathrm{i}\omega _j t} \big ] \otimes \big [ \sum _{\ell =1}^J \mathbf{G}_1(\mathrm{i}\omega _\ell ) e^{\mathrm{i}\omega _\ell t} \big ] \\&= \sum _{j=1}^J \mathbf{c}^T \mathbf{G}_1(\mathrm{i}\omega _j) e^{j \omega _j t} + \sum _{j=1}^J \sum _{\ell =1}^J \mathbf{K}\big [ \mathbf{G}_1(\mathrm{i}\omega _j) \otimes \mathbf{G}_1(\mathrm{i}\omega _\ell ) \big ] e^{\mathrm{i}(\omega _j + \omega _\ell )t}. \end{aligned} \end{aligned}$$

Substituting $\mathbf{G}_1(s) = (s\mathbf{I}_ {{\mathcal {N}}} -\mathbf{A})^{-1} \mathbf{b}$ back into the last equation yields the desired result (2.3) with $H_1(s)$ and $H_2(s,z)$ as defined in (2.4) and (2.5). $\square$

Lemma 2.1 shows that the LQO system (2.1) is characterized by two transfer functions, namely $H_1(s)$ (corresponding to the linear component $y_1(t)$ in the output) and $H_2(s,z)$ (corresponding to the quadratic component $y_2(t)$ in the output). As in the classical linear case, $H_1(s)$ is a rational function of a single variable. On the other hand, $H_2(s,z)$ is also a rational function, but of two variables. These two transfer functions that fully describe the LQO system (2.1) will play the fundamental role in our analysis to extend barycentric interpolation and AAA to the LQO setting. Before we establish the theory for LQO systems, we will briefly review the AAA algorithm for linear systems in Sect. 3.

#### Remark 2.1

In the proposed framework, we will require sampling the two transfer functions $H_1(s)$ and $H_2(s,z)$. This could be achieved by exciting the system (as a black box) with purely oscillatory signals (sines and cosines) as control inputs. Then, as shown in Lemma 2.1, measuring the steady-state part of the observed outputs corresponding to these inputs yields a linear combination between the required samples and complex exponentials. The two transfer function evaluations are, then, inferred from the harmonics of the output spectrum (in the frequency domain), by performing spectral (Fourier) transformations on the measured time-domain signals. For more details on such procedures in a similar setting, e.g., for inferring measurements of generalized transfer functions of bilinear systems, we refer the reader to [25]. We also note that [40] examines systems described by two time-domain kernels together with their Fourier transformations (deemed as transfer functions) and their measurements. Even though no explicit representation of these functions are considered in terms of a state-space realization, those ideas also equally apply to sample $H_1(s)$ and $H_2(s,z)$ as well.

#### Remark 2.2

For the special case of $\mathbf{K}= \alpha ( \mathbf{c}^T \otimes \mathbf{c}^T)$, we obtain $y_2(t) = \alpha y_1^2(t)$ where $\alpha$ is a scalar. Thus, in this case the output *y*(*t*) is a quadratic polynomial in the linear output $y_1(t)$ and the LQO model can be interpreted as a Wiener model [43]. However, our focus here is on general $\textsf {LQO}$ systems without this special case.

## Barycentric Rational Approximation for Linear Systems and the AAA Algorithm

For an underlying function $H(\cdot ): {{\mathbb {C}}}\rightarrow {{\mathbb {C}}}$, e.g., transfer function of a single-input/single-output (SISO) linear dynamical system, assume the following set of measurements:

3.1 $$\begin{aligned} \{H(s_i)\} \in {\mathbb {C}}~~\text{ where }~~s_i \in {\mathbb {C}}\quad \text{ for }~~ i=1,2,\ldots ,N_s. \end{aligned}$$

Partition the sampling points into two disjoint sets:3.2We will clarify later how this partitioning is chosen. Based on (3.2), define the sampled valuesand the corresponding data sets3.3Define the rational function *r*(*s*) in the barycentric form [11], a numerically stable representation of rational functions^1^:

3.4 $$\begin{aligned} r(s) = \dfrac{\mathfrak {p}(s)}{\mathfrak {q}(s)} = \frac{\displaystyle \sum _{k=1}^n \frac{w_k h_k}{s - {\xi }_k}}{\displaystyle 1+ \sum _{k=1}^n\frac{w_k}{s - {\xi }_k}}, \end{aligned}$$

where ${\xi }_k \in {\mathbb {C}}$ are the *sampling (support) points* and the *weights* $w_k \in {\mathbb {C}}$ are to be determined. By construction, the degree-*n* rational function *r*(*s*) in (3.4) is a rational interpolant at the support point set ${\varvec{{\xi }}}$, i.e.,

3.5 $$\begin{aligned} r({\xi }_k) = h_k \quad \text{ for }\quad k=1,2,\ldots ,n, \end{aligned}$$

assuming $w_k \ne 0$. Then, the freedom in choosing the weights $\{w_k\}$ can be used to match the remaining the data ${\widehat{\mathbf{h}}}$ in an appropriate measure.

Assuming enough degrees of freedom, [2] chooses the weights $\{w_k\}$ to enforce interpolation of ${\widehat{\mathbf{h}}}$ as well, by computing the null space of the corresponding divided difference matrix, thus obtaining a degree-*n* rational function interpolating the full data (3.1). We skip the details for the conditions to guarantee the existence and uniqueness of such a rational interpolant and refer the reader to [2, 3] for details.

The Adaptive Antoulas-Anderson (AAA) algorithm [32], on the other hand, elegantly combines interpolation and least-squares (LS) fitting. In the barycentric form (3.4), which interpolates the data $\mathbf{h}$ by construction, AAA chooses the weights $\{w_k\}$ to minimize a LS error over the data ${\widehat{\mathbf{h}}}$. Note that the LS problem over ${\widehat{\mathbf{h}}}$ is nonlinear in the weights $\{w_k\}$ since these weights appear in the denominator of *r*(*s*) as well. AAA solves a relaxed linearized LS problem instead. For a sampling point ${\widehat{{\xi }}}_i$ in the set ${\varvec{{\xi }}}$, AAA uses the linearization

3.6 $$\begin{aligned} {\widehat{h}}_i - r({\widehat{{\xi }}}_i) = \dfrac{1}{\mathfrak {q}({\widehat{{\xi }}}_i)} \left( {\widehat{h}}_i \mathfrak {q} ({\widehat{{\xi }}}_i) - \mathfrak {p} ({\widehat{{\xi }}}_i) \right) \rightsquigarrow {\widehat{h}}_i \mathfrak {q} ({\widehat{{\xi }}}_i) - \mathfrak {p} ({\widehat{{\xi }}}_i), \end{aligned}$$

leading to the linearized LS problem

3.7 $$\begin{aligned} \min _{w_1,\ldots ,w_{k}} \sum _{i=1}^{N_s-n} \mid {\widehat{h}}_i \mathfrak {q}({\widehat{{\xi }}}_i) - \mathfrak {p}({\widehat{{\xi }}}_i) \mid ^2. \end{aligned}$$

AAA is an iterative algorithm and builds the partitioning (3.2) using a greedy search. Assume in step *n*, AAA has the rational approximant *r*(*s*) as in (3.4) corresponding to the partitioning (3.2) where the weights $\{w_k\}$ are selected by solving (3.7). AAA updates (3.2) via a greedy search by finding ${\widehat{{\xi }}}_i \in {\widehat{{\varvec{{\xi }}}}}$ for which the error $\mid r({\widehat{{\xi }}}_i) - {\widehat{h}}_i\mid$ is the largest. This sampling point is then added to the interpolation set ${\varvec{{\xi }}}$, the barycentric rational approximant *r*(*s*) in (3.4) is updated accordingly (it has one higher degree now), and the new weights are computed, as before, by solving a linearized LS problem. The procedure is repeated until either a desired order or an error tolerance is obtained. For further details, we refer the reader to the original source [32]. The AAA algorithm proved very flexible and effective, and has been employed in various applications such as rational approximation over disconnected domains [32], solving nonlinear eigenvalue problems [28], modeling of parametrized dynamics [13], and approximation of matrix-valued functions [20].

## Barycentric Representations for LQO Systems

To develop interpolating barycentric forms for $H_1(s)$ and $H_2(s,z)$, we first need to specify the data corresponding to the underlying LQO system $\Sigma _{\textsf {LQO}}$. The first transfer function $H_1(s)$ of $\Sigma _{\textsf {LQO}}$ is a single-variable rational function and, as in Sect. 3, we sample $H_1(s)$ at distinct points $\{s_1,\ldots ,s_{N_s}\}$ to obtain the data set

4.1 $$\begin{aligned} \{H_1(s_i)\} \in {\mathbb {C}}~~\text{ where }~~s_i \in {\mathbb {C}}\quad \text{ for }~~ i=1,2,\ldots ,N_s. \end{aligned}$$

The second transfer function $H_2(s,z)$, on the other hand, is a function of two-variables. Therefore, in agreement with the data (4.1), we will sample $H_2(s,z)$ at the corresponding rectangular grid: for $i,j=1,2,\ldots ,N_s$,

4.2 $$\begin{aligned} \{H_2(s_i,s_j)\} \in {\mathbb {C}}~~\text{ where }~~s_i,s_j \in {\mathbb {C}}. \end{aligned}$$

Partition the full set of sampling points into two disjoint sets

4.3 $$\begin{aligned} \{ s_1, \dots , s_{N_s} \} = \{~{\xi }_1, \dots , {\xi }_{n} ~\} \cup \{~{\widehat{{\xi }}}_1, \dots , {\widehat{{\xi }}}_{N_s-n}~\} = {\varvec{{\xi }}}~ \cup ~ {\widehat{{\varvec{{\xi }}}}}\end{aligned}$$

and define the sampled values (measurements):4.4and4.5Then, the goal is to a construct a *data-driven* LQO system directly from these samples without access to the internal dynamics of $\Sigma _{\textsf {LQO}}$. The partition (4.3) and the error measure used in approximating the data will be clarified later. First we will show how the data in (4.1) and (4.2) can be used to develop barycentric-like representations corresponding to an LQO system. We will use the notation $r_1(s)$ to denote the rational approximation to $H_1(s)$ and $r_2(s,z)$ to $H_2(s,z)$.

### Proposition 4.1

Given the $H_1(s)$ samples in (4.1), pick the nonzero barycentric weights $\{w_1,w_2,\ldots ,w_n\}$. Then, the barycentric rational function

4.6 $$\begin{aligned} r_1(s) = \dfrac{{\mathfrak {p}_1(s)}}{{\mathfrak {q}_1(s)}} = {\displaystyle \sum _{k=1}^n \frac{ w_k h_k }{s - {\xi }_k }} \Bigg /\left( {1+\displaystyle \sum _{k=1}^n \frac{w_k }{s- {\xi }_k}}\right) \end{aligned}$$

interpolates the data in (4.4). Let $\mathbf{e}\in {\mathbb {C}}^n$ denote the vector of ones. Define

4.7 $$\begin{aligned} \begin{aligned} \widehat{\mathbf{b}}&= \left[ \begin{array}{cccc} w_1&w_2&\ldots&w_n \end{array} \right] ^T \in {\mathbb {C}}^n,\qquad \varvec{\Xi }= {\mathsf {diag}}({\xi }_1,\ldots ,{\xi }_n) \in {\mathbb {C}}^{n\times n}, \\ \widehat{\mathbf{A}}&= \varvec{\Xi } - \widehat{\mathbf{b}}\mathbf{e}^T\in {\mathbb {C}}^{n\times n}, \qquad \text{ and }~\qquad \widehat{\mathbf{c}}^T = \left[ \begin{array}{cccc} h_1&h_2&\ldots&h_n \end{array} \right] \in {\mathbb {C}}^{n}. \end{aligned} \end{aligned}$$

Then, $r_1(s)$ has the state-space form

4.8 $$\begin{aligned} r_1(s)= \widehat{\mathbf{c}}^T(s{\hat{\mathbf{I}}}_n-\widehat{\mathbf{A}})^{-1}\widehat{\mathbf{b}}, \end{aligned}$$

where ${\hat{\mathbf{I}}}_n$ is the identity matrix of dimension $n\times n$.

### Proof

The fact that $r_1(s)$ is an interpolating rational function for the data (4.4) is just a restatement of (3.5) for completeness. To prove (4.8), we will use the *Sherman-Morrison* formula [18]: Let $\mathbf{M}\in {\mathbb {C}}^{n \times n}$ be invertible and $\mathbf{u},\mathbf{v}\in {\mathbb {C}}^n$ be such that $1 + \mathbf{v}^* \mathbf{M}^{-1} \mathbf{u}\ne 0$ where $(\cdot )^*$ denotes the conjugate transpose. Then,

4.9 $$\begin{aligned} (\mathbf{M}+ \mathbf{u}\mathbf{v}^*) ^ {-1} = \mathbf{M}^{-1} - \frac{\mathbf{M}^{-1} \mathbf{u}\mathbf{v}^* \mathbf{M}^{-1}}{ 1+\mathbf{v}^* \mathbf{M}^{-1} \mathbf{u}}. \end{aligned}$$

From (4.7) and (4.8), we have

4.10 $$\begin{aligned} r_1(s) = \widehat{\mathbf{c}}^T (s {{\hat{\mathbf{I}}}}_n - \widehat{\mathbf{A}})^{-1} \widehat{\mathbf{b}}= \widehat{\mathbf{c}}^T [(s {{\hat{\mathbf{I}}}}_n - \varvec{\Xi })+\widehat{\mathbf{b}}\mathbf{e}^T]^{-1} \widehat{\mathbf{b}}. \end{aligned}$$

To simplify the notation, let ${\hat{ \varvec{\Phi } }}_{s} = s {{\hat{\mathbf{I}}}}_n - \widehat{\varvec{A}}$. Then, applying the Sherman-Morrison formula to the middle term in (4.10) with $\mathbf{M}={\hat{ \varvec{\Phi } }}_{s}$, $\mathbf{u}= \widehat{\mathbf{b}}$, and $\mathbf{v}= \mathbf{e}$, we obtain

4.11 $$\begin{aligned} r_1(s)&= \widehat{\mathbf{c}}^T \left( {\hat{ \varvec{\Phi } }}_{s}+\widehat{\mathbf{b}}\mathbf{e}^T\right) ^{-1} \widehat{\mathbf{b}}\nonumber \\&= \widehat{\mathbf{c}}^T \left( {\hat{ \varvec{\Phi } }}_s^{-1} - \frac{{\hat{ \varvec{\Phi } }}_{s}^{-1} \widehat{\mathbf{b}}\mathbf{e}^T {\hat{ \varvec{\Phi } }}_s^{-1}}{ 1+\mathbf{e}^T {\hat{ \varvec{\Phi } }}_{s}^{-1} \widehat{\mathbf{b}}} \right) \widehat{\mathbf{b}}\nonumber \\&= \widehat{\mathbf{c}}^T \left( {\hat{ \varvec{\Phi } }}_{s}^{-1} \widehat{\mathbf{b}}- \frac{ {\hat{ \varvec{\Phi } }}_{s}^{-1} \widehat{\mathbf{b}}\cdot \mathbf{e}^T {\hat{ \varvec{\Phi } }}_{s}^{-1} \widehat{\mathbf{b}}}{1+\mathbf{e}^T {\hat{ \varvec{\Phi } }}_{s}^{-1} \widehat{\mathbf{b}}} \right) = \widehat{\mathbf{c}}^T\frac{ {\hat{ \varvec{\Phi } }}_{s}^{-1} \widehat{\mathbf{b}}}{1+\mathbf{e}^T {\hat{ \varvec{\Phi } }}_{s}^{-1} \widehat{\mathbf{b}}}. \end{aligned}$$

Since $\varvec{\Xi }$ is diagonal,

$$\begin{aligned} {\hat{ \varvec{\Phi } }}_{s}^{-1} = (s {{\hat{\mathbf{I}}}}_n - \varvec{\Xi })^{-1} = \mathsf {diag}(\left[ \begin{array}{ccc} (s - {\xi }_1)^{-1}&\ldots&(s - {\xi }_n)^{-1} \end{array} \right] ). \end{aligned}$$

Then, using the definitions of $\widehat{\mathbf{b}}$ and $\widehat{\mathbf{c}}$ in (4.7), we obtain

4.12 $$\begin{aligned} \widehat{\mathbf{c}}^T{\hat{ \varvec{\Phi } }}_{s}^{-1} \widehat{\mathbf{b}}= \displaystyle \sum _{k=1}^n \frac{ w_k h_k }{s - {\xi }_k } ~~\text{ and }~~ \mathbf{e}^T {\hat{ \varvec{\Phi } }}_{s}^{-1} \widehat{\mathbf{b}}= \displaystyle \sum _{k=1}^n \frac{ w_k}{s - {\xi }_k }. \end{aligned}$$

Substituting these last two equalities into (4.11) yields (4.8). $\square$

We note that state-space realizations for rational functions are unique up to a similarity transformation. For other equivalent state-space representations of a barycentric form, we refer the reader to, e.g., [5, 28].

Given the samples of $H_1(s)$ (data in (4.4)) of the LQO system (2.1), Proposition 4.1 constructs the *linear* part of the data-driven LQO model, directly from these samples. What we need to achieve next is to use the $H_2(s,z)$ samples (data in (4.5)) to construct a two-variable rational function $r_2(s,z)$ in a barycentric-like form corresponding to the quadratic part of the data-driven LQO model. However, $r_2(s,z)$ *cannot be constructed independently* from $r_1(s)$. Once $r_2(s,z)$ is constructed, we should be able to interpret $r_1(s)$ and $r_2(s,z)$ together as the linear and quadratic transfer functions of a single LQO system. This is the precise reason why we cannot simply view $r_2(s,z)$ as an independent two-variable rational function and use the classical multivariate barycentric form [3, 4]. Therefore, $r_2(s,z)$ needs to have the form

$$\begin{aligned} r_2(s,z) = {\hat{\mathbf{K}}} \big [ (s{\hat{\mathbf{I}}}-\widehat{\mathbf{A}})^{-1} \widehat{\mathbf{b}}\otimes (z{\hat{\mathbf{I}}}-\widehat{\mathbf{A}}) \widehat{\mathbf{b}}\big ], \end{aligned}$$

where $\widehat{\mathbf{A}}$ and $\widehat{\mathbf{b}}$ are the same matrices from (4.7) used in modeling $r_1(s)$ and ${\hat{\mathbf{K}}} \in {{\mathbb {C}}}^{1 \times n^2}$ is the (quadratic) free variable that will incorporate to model the new data (4.5). The next result achieves this goal.

### Theorem 4.1

Assume the set-up in Proposition 4.1 and that the samples of $H_2(s,z)$ in (4.2) are given. Define the two-variable function $r_2(s,z)$ in a barycentric-like form:

4.13 $$\begin{aligned} r_2(s,z) = \frac{\displaystyle \sum _{k=1}^n \sum _{\ell =1}^n \frac{ h_{k,\ell }w_k w_\ell }{(s-{\xi }_k)(z-{\xi }_\ell )}}{1+\displaystyle \sum _{k=1}^n \frac{w_k }{s- {\xi }_k} + \displaystyle \sum _{\ell =1}^n \frac{w_\ell }{z- {\xi }_\ell } +\displaystyle \sum _{k=1}^n \sum _{\ell =1}^n \frac{ w_k w_\ell }{(s-{\xi }_k)(z-{\xi }_\ell )}}. \end{aligned}$$

Then, $r_2(s,z)$ interpolates the data (4.5), i.e.,

4.14 $$\begin{aligned} r_2({\xi }_i,{\xi }_j) = H_2({\xi }_i,{\xi }_j)~~\text{ for } ~~i,j = 1,\ldots ,n. \end{aligned}$$

Define ${\hat{\mathbf{M}}} \in {\mathbb {C}}^{n \times n}$ and ${\hat{\mathbf{K}}} \in {\mathbb {C}}^{1 \times n^2}$ using

4.15 $$\begin{aligned}{}[{\hat{\mathbf{M}}}]_{i,j} = h_{i,j}~~\text{ for }~~i,j=1,2,\ldots ,n \quad \text{ and } \quad {\hat{\mathbf{K}}} = [\mathsf {vec}(\hat{\mathbf{M})}]^T. \end{aligned}$$

Then, $r_2(s,z)$ has the state-space form

4.16 $$\begin{aligned} r_2(s,z) = {\hat{\mathbf{K}}} \big [ (s{\hat{\mathbf{I}}}-\widehat{\mathbf{A}})^{-1} \widehat{\mathbf{b}}\otimes (z{\hat{\mathbf{I}}}-\widehat{\mathbf{A}})^{-1} \widehat{\mathbf{b}}\big ]. \end{aligned}$$

### Proof

To prove the interpolation property (4.14) of the barycentric representation (4.13), we start by introducing various polynomials in one or two variables:

4.17 $$\begin{aligned} \begin{aligned} \mathfrak {m}(s)&= \prod _{k=1}^n (s-\xi _k), \ \  \mathfrak {M}(s,z) = \prod _{k=1}^n \prod _{\ell =1}^n (s-\xi _k)(z-\xi _\ell ), \\ \mathfrak {m}_i(s)&= \prod _{k=1,k \ne i}^n (s-\xi _k), \ \ ~\text{ and }~~ \mathfrak {M}_{i,j}(s,z) = \prod _{k=1, k \ne i}^n \prod _{\ell =1, \ell \ne j}^n (s-\xi _k) (z-\xi _\ell ), \end{aligned} \end{aligned}$$

for $i,j=1,\ldots ,n$. Multiply both the numerator and denominator of $r_2(s,z)$ in (4.13) with $\mathfrak {M}(s,z)$ to obtain

4.18 $$\begin{aligned} r_2(s,z)&= \frac{\mathfrak {p}_2(s,z)}{\mathfrak {q}_2(s,z)}, \end{aligned}$$

with

4.19 $$\begin{aligned} \begin{aligned} \mathfrak {p}_2(s,z)&= \displaystyle \sum _{k=1}^n \sum _{\ell =1}^n h_{k,\ell }w_k w_\ell \mathfrak {M}_{k,\ell }(s,z),~~\text{ and } \\ \mathfrak {q}_2(s,z)&= \mathfrak {M}(s,z)+\displaystyle \sum _{k=1}^n w_k \mathfrak {m}_k(s) \mathfrak {m}(z) + \displaystyle \sum _{\ell =1}^n w_\ell \mathfrak {m}_\ell (z) \mathfrak {m}(s) +\displaystyle \sum _{k=1}^n \sum _{\ell =1}^n w_k w_\ell \mathfrak {M}_{k,\ell }(s,z). \end{aligned} \end{aligned}$$

Then, evaluate $r_2(s,z)$ at $s = \xi _i$ and $z = \xi _j$ to obtain

$$\begin{aligned} r_2(\xi _i,\xi _j) = \frac{\mathfrak {p}_2(\xi _i,\xi _j)}{\mathfrak {q}_2(\xi _i,\xi _j)} = \frac{h_{i,j}w_i w_j \mathfrak {M}_{i,j}(\xi _i,\xi _j)}{w_i w_j \mathfrak {M}_{i,j}(\xi _i,\xi _j)} = h_{i,j}. \end{aligned}$$

To prove (4.16), we first note that

$$\begin{aligned} \begin{aligned} r_2(s,z)&= {\hat{\mathbf{K}}} \left[ (s{\hat{\mathbf{I}}}_n-\widehat{\mathbf{A}})^{-1} \widehat{\mathbf{b}}\otimes (z{\hat{\mathbf{I}}}_n-\widehat{\mathbf{A}})^{-1} \widehat{\mathbf{b}}\right] = {{\hat{\mathbf{K}}}} \left[ \frac{ {\hat{ \varvec{\Phi } }}_{s}^{-1} \widehat{\mathbf{b}}}{1+\mathbf{e}^T {\hat{ \varvec{\Phi } }}_{s}^{-1} \widehat{\mathbf{b}}} \otimes \frac{ {\hat{ \varvec{\Phi } }}_{z}^{-1} \widehat{\mathbf{b}}}{1+\mathbf{e}^T {\hat{ \varvec{\Phi } }}_{z}^{-1} \widehat{\mathbf{b}}} \right] , \end{aligned} \end{aligned}$$

where we used the fact

$$\begin{aligned} (s{{\hat{\mathbf{I}}}}_n - \widehat{\mathbf{A}})^{-1} \widehat{\mathbf{b}}= \left( {\hat{ \varvec{\Phi } }}_{s}+\widehat{\mathbf{b}}\mathbf{e}^T\right) ^{-1}\widehat{\mathbf{b}}= \frac{ {\hat{ \varvec{\Phi } }}_{s}^{-1} \widehat{\mathbf{b}}}{1+\mathbf{e}^T {\hat{ \varvec{\Phi } }}_{s}^{-1} \widehat{\mathbf{b}}}, \end{aligned}$$

as shown in deriving (4.11). Since ${\hat{ \varvec{\Phi } }}_{s}$ diagonal, we have

4.20 $$\begin{aligned} r_2(s,z) = \frac{{{\hat{\mathbf{K}}}}}{{(1+\mathbf{e}^T {\hat{ \varvec{\Phi } }}_{s} \widehat{\mathbf{b}})( 1+\mathbf{e}^T {\hat{ \varvec{\Phi } }}_{z} \widehat{\mathbf{b}})}} \left[ \begin{array}{c} \frac{w_1}{s-{\xi }_1} \\ \vdots \\ \frac{w_n}{s-{\xi }_n} \end{array} \right] \otimes \left[ \begin{array}{c} \frac{w_1}{z-{\xi }_1} \\ \vdots \\ \frac{w_n}{z-{\xi }_n} \end{array} \right] . \end{aligned}$$

Then, substituting into (4.20) the definition of ${{\hat{\mathbf{K}}}}$ from (4.15) and the second formula in (4.12), we obtain

4.21 $$\begin{aligned} r_2(s,z)&= \frac{\sum _{k=1}^n \sum _{\ell =1}^n \frac{h_{k,\ell } w_k w_\ell }{(s-{\xi }_k)(z-{\xi }_\ell )}}{\Big (1+\displaystyle \sum _{k=1}^n \frac{w_k }{s- {\xi }_k}\Big )\Big (1+ \sum _{\ell =1}^n \frac{w_\ell }{z- {\xi }_\ell }\Big )} \nonumber \\&= \frac{\displaystyle \sum _{k=1}^n \sum _{\ell =1}^n \frac{h_{k,\ell } w_k w_\ell }{(s-{\xi }_k)(z-{\xi }_\ell )}}{1+\displaystyle \sum _{k=1}^n \frac{w_k }{s- {\xi }_k} + \sum _{\ell =1}^n \frac{w_\ell }{z- {\xi }_\ell } + \sum _{k=1}^n \sum _{\ell =1}^n \frac{ w_k w_\ell }{(s-{\xi }_k)(z-{\xi }_\ell )}}, \end{aligned}$$

which concludes the proof. $\square$

The next result directly follows from Proposition 4.1 and Theorem 4.1.

### Corollary 4.1

Assume the set-ups in Proposition 4.1 and Theorem 4.1. Then, interpolating rational functions $r_1(s)$ and $r_2(s,z)$ jointly correspond to an interpolatory LQO model

4.22 $$\begin{aligned} \widehat{\Sigma }_{\textsf {LQO}}: {\left\{ \begin{array}{ll} \dot{{\hat{\mathbf{x}}}}(t)=\widehat{\mathbf{A}}\mathbf{x}(t)+\widehat{\mathbf{b}}u(t),\\  {\hat{y}}(t)=\widehat{\mathbf{c}}^T{\hat{\mathbf{x}}}(t) +{{\hat{\mathbf{K}}} \big [ {\hat{\mathbf{x}}}(t) \otimes {\hat{\mathbf{x}}}(t) \big ]}. \end{array}\right. } \end{aligned}$$

In others words, the first (linear) transfer function of $\widehat{\Sigma }_{\textsf {LQO}}$ is $r_1(s)$ and its second transfer function is $r_2(s,z)$.

Recall the partitioning of sampling points in (4.3). Theorem 4.1 has shown that $r_2(s,z)$ interpolates $H_2(s,z)$ over the sampling set ${\varvec{{\xi }}}\times {\varvec{{\xi }}}$. What is the value of $r_2(s,z)$ over the *mixed* sampling sets ${\varvec{{\xi }}}\times {\widehat{{\varvec{{\xi }}}}}$ and ${\widehat{{\varvec{{\xi }}}}}\times {\varvec{{\xi }}}$? Even though we do not enforce interpolation over these sets, in Sect. 5 we will need a closed-form expression for the value of $r_2(s,z)$ over ${\varvec{{\xi }}}\times {\widehat{{\varvec{{\xi }}}}}$ and ${\widehat{{\varvec{{\xi }}}}}\times {\varvec{{\xi }}}$. The next lemma establishes these results.

### Lemma 4.1

Let $r_2(s,z)$ be as defined in (4.13) corresponding to the sampling points in (4.3) and the data in (4.2). Then,

4.23 $$\begin{aligned} r_2({\xi }_i,{\widehat{{\xi }}}_j)&= \frac{\displaystyle \sum _{\ell =1}^n \frac{w_\ell h_{i,\ell } }{{\widehat{{\xi }}}_j-{\xi }_\ell }}{1+\displaystyle \sum _{\ell =1}^n \frac{ w_\ell }{{\widehat{{\xi }}}_j-{\xi }_\ell }} \ \ ~\text{ and } ~\ \ r_2({\widehat{{\xi }}}_j,{\xi }_i) = \frac{\displaystyle \sum _{k=1}^n \frac{w_k h_{k,i} }{{\widehat{{\xi }}}_j-{\xi }_k}}{1+\displaystyle \sum _{k=1}^n \frac{ w_k}{{\widehat{{\xi }}}_j-{\xi }_k}} \end{aligned}$$

for $$i = 1,\ldots , n$$ and $$j = 1, \ldots , N_s-n$$.

### Proof

Proof is given in at the end of the paper. $\square$

It is important to note that the numerators and denominators of $r_2({\xi }_i,{\widehat{{\xi }}}_j)$ and $r_2({\widehat{{\xi }}}_j,{\xi }_i)$ in (4.23) are *linear* in the weights $w_\ell$. This is in contrast to the general form of $r_2(s,z)$ in (4.21) where both the numerator and denominator are quadratic in $w_\ell$ when evaluated over ${\widehat{{\varvec{{\xi }}}}}\times {\widehat{{\varvec{{\xi }}}}}$.

## Proposed Framework for Data-Driven Modeling of LQO Systems

Section 4 established the necessary ingredients to extend AAA to LQO systems. Given the measurements (4.1) and (4.2), Proposition 4.1 and Theorem 4.1 show how to construct the barycentric forms $r_1(s)$ and $r_2(s,z)$ interpolating this data in accordance with the partitioning (4.3). Furthermore, Corollary 4.1 states that $r_1(s)$ and $r_2(s,z)$ together correspond to an interpolatory LQO system. Based on these results, we will now fully develop the AAA framework for LQO systems. The resulting algorithm will be denoted by AAA-LQO.

AAA-LQO will be an iterative algorithm, adding one degree of freedom to the current data-driven LQO model in every iteration step. To emphasize the iterative nature of the algorithm, in the *n*th step of AAA-LQO, we will use the notation $r_1^{(n)}(s)$ and $r_2^{(n)}(s,z)$ to represent a data-driven order-*n* LQO model for the partitioning in (4.3). First, for this *current* partitioning, in Sect. 5.1, we introduce a LS error measure to determine the barycentric weights $\{w_k\}$ appearing in the definitions of $r_1^{(n)}(s)$ and $r_2^{(n)}(s,z)$ in (4.6) and (4.13). Then, in Sect. 5.2 we establish a greedy search procedure for updating the partitioning (4.3). The algorithm will then continue with the LS minimization for the updated partitioning at the $(n+1)$th step to construct $r_1^{(n+1)}(s)$ and $r_2^{(n+1)}(s,z)$. AAA-LQO will terminate after a desired error criterion is met or a maximum allowed order is achieved as explained in Sect. 5.3.

### A Combined LS Measure for Computing the Barycentric Weights for the Current Partition

Even though this section introduces and investigates the LS problem in the *n*th step of AAA-LQO, to simplify the notation for the complicated expressions appearing in the analysis, we will drop the superscript *n* and use $r_1(s)$ and $r_2(s,z)$ instead (as we did in Sect. 4). However, they should be understood as the approximants in the *n*th step. We will reintroduce the superscripts in Sect. 5.2.

For the full LQO data (4.1) and (4.2), we recall (and repeat) the partitioning of the sampling points as in (4.3):

5.1 $$\begin{aligned} \{ s_1, \dots , s_{N_s} \} = \{~{\xi }_1, \dots , {\xi }_{n} ~\} \cup \{~{\widehat{{\xi }}}_1, \dots , {\widehat{{\xi }}}_{N_s-n}~\} = {\varvec{{\xi }}}~\cup ~ {\widehat{{\varvec{{\xi }}}}}. \end{aligned}$$

Then, $r_1(s)$ interpolates $H_1(s)$ over ${\varvec{{\xi }}}$ (i.e., it interpolates the data (4.4)) and $r_2(s,z)$ interpolates $H_2(s,z)$ over ${\varvec{{\xi }}}\times {\varvec{{\xi }}}$ (i.e., it interpolates the data (4.5)). Also together, $r_1(s)$ and $r_2(s,z)$ correspond to an LQO system. The only remaining degrees of freedom in defining $r_1(s)$ and $r_2(s,z)$, and thus the corresponding LQO system, are the barycentric weights $\{w_1,\ldots ,w_n\}$. We will choose those weights to minimize an appropriate error measure in the *uninterpolated* data corresponding to the sampling points ${\widehat{{\varvec{{\xi }}}}}$. We first introduce the notation for these *uninterpolated* values:^2^5.25.35.45.5Let $\mathbf{w}\in {\mathbb {C}}^n$ denote the vector of weights to be determined:

$$\begin{aligned} \mathbf{w}= \begin{bmatrix} w_1 \\ w_2 \\ \vdots \\ w_n \end{bmatrix}. \end{aligned}$$

A reasonable error measure to minimize is the LS distance in the uninterpolated data, leading to the minimization problem

5.6 $$\begin{aligned} \min \limits _{\mathbf{w}\ne \mathbf {0}} \left( {{\mathcal {J}}}_1+ {{\mathcal {J}}}_2 + {{\mathcal {J}}}_3 + {{\mathcal {J}}}_4\right) , \end{aligned}$$

where

5.7 $$\begin{aligned} {{\mathcal {J}}}_1&= {\frac{1}{N_s-n}}\sum _{i=1}^{N_s-n} (r_1({\widehat{{\xi }}}_i) - {\widehat{h}}_i)^2, \end{aligned}$$

5.8 $$\begin{aligned} {{\mathcal {J}}}_2&={\frac{1}{n(N_s-n)}} \sum _{i=1}^{n} \sum _{j=1}^{N_s-n} (r_2({\xi }_i,{\widehat{{\xi }}}_j) -{\widehat{h}}_{i,j}^{{\mathrm{(1,2)}}})^2, \end{aligned}$$

5.9 $$\begin{aligned} {{\mathcal {J}}}_3&= {\frac{1}{(N_s-n)n}} \sum _{i=1}^{N_s-n} \sum _{j=1}^{n} (r_2({\widehat{{\xi }}}_i,{\xi }_j) - {\widehat{h}}_{j,i}^{{\mathrm{(2,1)}}})^2,~\text{ and } \end{aligned}$$

5.10 $$\begin{aligned} {{\mathcal {J}}}_4&= {\frac{1}{(N_s-n)^2}} \sum _{i=1}^{N_s-n} \sum _{j=1}^{N_s-n} (r_2({\widehat{{\xi }}}_i,{\widehat{{\xi }}}_j) - {\widehat{h}}_{i,j}^{{\mathrm{(2,2)}}})^2. \end{aligned}$$

As in the original AAA for linear dynamical systems, the LS problem (5.6) is nonlinear in $\mathbf{w}$ for LQO systems. The formulation is more complicated here due to the additional $r_2(s,z)$ term. To resolve this numerical difficulty, we will employ a strategy, similar to the lineraziation step in (3.6), and solve a relaxed optimization problem. However, the resulting LS problem in our case will still be nonlinear, yet much easier to solve than (5.6). In the end, we will tackle the original nonlinear LS problem (5.6) by solving a sequence of quadratic LS problems. We note that in (5.7)–(5.10), we scale every error term ${{\mathcal {J}}}_i$ with the number of data points in it.

#### Quadraticized LS Problem in Step *n*

We show how to relax each ${{\mathcal {J}}}_i$ term in the nonlinear LS problem (5.6). The resulting problem will play a crucial role in the proposed iterative algorithm (Sect. 5.3).

*Linearizing* ${{\mathcal {J}}}_1$ The *i*th term of ${{\mathcal {J}}}_1$ in (5.7), namely $r_1({\widehat{{\xi }}}_i) - {\widehat{h}}_i$, is the same as in (3.6). This is natural since $r_1(s)$ corresponds to the linear part of the LQO system. Thus, we linearize ${{\mathcal {J}}}_1$ similar to (3.6). Write $r_1(s)$ as $r_1(s) = \mathfrak {p}_1(s)/\mathfrak {q}_1(s)$, as defined in (4.6). Then, the *i*th term in (5.7) is linearized as

5.11 $$\begin{aligned} r_1({\widehat{{\xi }}}_i) - {\widehat{h}}_i = \dfrac{1}{\mathfrak {q}_1({\widehat{{\xi }}}_i)} \left( \mathfrak {p}_1({\widehat{{\xi }}}_i) - {\widehat{h}}_i \mathfrak {q}_1 ({\widehat{{\xi }}}_i) \right) \rightsquigarrow \mathfrak {p}_1 ({\widehat{{\xi }}}_i)- {\widehat{h}}_i \mathfrak {q}_1 ({\widehat{{\xi }}}_i). \end{aligned}$$

Substituting $\mathfrak {p}_1(s)$ and $\mathfrak {q}_1(s)$ from the definition of $r_1(s)$ in (4.6) into (5.11), one obtains

5.12 $$\begin{aligned} \mathfrak {p}_1({\widehat{{\xi }}}_i)- {\widehat{h}}_i \mathfrak {q}_1({\widehat{{\xi }}}_i)&= \sum _{k=1}^n \frac{ w_k h_k }{{\widehat{{\xi }}}_i - {\xi }_k } - {\widehat{h}}_i \left( 1+ \sum _{k=1}^n \frac{w_k }{{\widehat{{\xi }}}_i- {\xi }_k} \right) =\sum _{k=1}^n \frac{w_k(h_k-{\widehat{h}}_i)}{{\widehat{{\xi }}}_i- {\xi }_k} - {\widehat{h}}_i. \end{aligned}$$

For a matrix $\mathbf{X}$, let $\left( \mathbf{X}\right) _{ij}$ denote its (*ij*)th entry. Similarly, for a vector $\mathbf{x}$, let $\left( \mathbf{x}\right) _{i}$ denote its *i*th entry. Define the Loewner matrix ${{\mathbb {L}}}\in {\mathbb {C}}^{(N_s-n)\times n}$ with

5.13 $$\begin{aligned} \left( {{\mathbb {L}}}\right) _{ik} = \frac{{\widehat{h}}_i-h_k}{{\widehat{{\xi }}}_i- {\xi }_k},~~\text{ for }~~ i=1,\ldots ,N_s,~k=1,\ldots ,n, \end{aligned}$$

and the vector ${\widehat{\mathbf{h}}}\in {\mathbb {C}}^{N_s-n}$ with $\left( {\widehat{\mathbf{h}}}\right) _i = {\widehat{h}}_i$. Then,

$$\begin{aligned} \sum _{i=1}^{N_s-n} \left( \mathfrak {p}_1 ({\widehat{{\xi }}}_i)- {\widehat{h}}_i \mathfrak {q}_1 ({\widehat{{\xi }}}_i) \right) ^2 = \Vert {{\mathbb {L}}}\mathbf{w}+ {\widehat{\mathbf{h}}}\Vert _2^2. \end{aligned}$$

Therefore, the ${{\mathcal {J}}}_1$ term in (5.7) will be relaxed to

5.14 $$\begin{aligned} {{\mathcal {J}}}_1 \rightsquigarrow {\frac{1}{N_s-n}} \Vert {{\mathbb {L}}}\mathbf{w}+ {\widehat{\mathbf{h}}}\Vert _2^2. \end{aligned}$$

*Linearizing* ${{\mathcal {J}}}_2$ *and* ${{\mathcal {J}}}_3$ Now we extend the linearization strategy used in ${{\mathcal {J}}}_1$, which only involved the single-variable function $r_1(s)$, to the error terms ${{\mathcal {J}}}_2$ and ${{\mathcal {J}}}_3$, which involve $r_2(s,z)$. The closed-form expressions for $r_2({\xi }_i,{\widehat{{\xi }}}_j)$ and $r_2({\widehat{{\xi }}}_j,{\xi }_i)$ we derived in Lemma 4.1 will prove fundamental in achieving these goals.

We start with ${{\mathcal {J}}}_2$. Write $r_2(s,z) = \mathfrak {p}_2(s,z)/\mathfrak {q}_2(s,z)$ as in (4.18). Then, linearizing the (*ij*)th term in (5.8) means

5.15 $$\begin{aligned} r_2({\xi }_i,{\widehat{{\xi }}}_j) - {\widehat{h}}_{i,j}^{{\mathrm{(1,2)}}}&= \dfrac{1}{\mathfrak {q}_2({\xi }_i,{\widehat{{\xi }}}_j)} \left( \mathfrak {p}_2({\xi }_i,{\widehat{{\xi }}}_j) - {\widehat{h}}_{i,j}^{{\mathrm{(1,2)}}}\mathfrak {q}_1 ({\xi }_i,{\widehat{{\xi }}}_j) \right) \nonumber \\&\rightsquigarrow \mathfrak {p}_2 ({\xi }_i,{\widehat{{\xi }}}_j)- {\widehat{h}}_{i,j}^{{\mathrm{(1,2)}}}\mathfrak {q}_2 ({\xi }_i,{\widehat{{\xi }}}_j). \end{aligned}$$

We substitute $\mathfrak {p}_2({\xi }_i,{\widehat{{\xi }}}_j)$ and $\mathfrak {q}_2({\xi }_i,{\widehat{{\xi }}}_j)$ from (4.23) into (5.15) to obtain

5.16 $$\begin{aligned} \mathfrak {p}_2 ({\xi }_i,{\widehat{{\xi }}}_j) - {\widehat{h}}_{i,j}^{{\mathrm{(1,2)}}}\mathfrak {q}_2 ({\xi }_i,{\widehat{{\xi }}}_j)&= \sum _{\ell =1}^n \frac{w_\ell h_{i,\ell } }{{\widehat{{\xi }}}_j-{\xi }_\ell } - {\widehat{h}}_{i,j}^{{\mathrm{(1,2)}}}\left( {1+ \sum _{\ell =1}^n \frac{ w_\ell }{{\widehat{{\xi }}}_j-{\xi }_\ell }}\right) \nonumber \\&= -\left( \sum _{\ell =1}^n \frac{ w_\ell ({\widehat{h}}_{i,j}^{{\mathrm{(1,2)}}}-h_{i,\ell })}{{\widehat{{\xi }}}_j- {\xi }_\ell } + {\widehat{h}}_{i,j}^{{\mathrm{(1,2)}}}\right) . \end{aligned}$$

Define the indexing variable $\alpha _{ij} = (i-1)(N_s-n)+j$ and let $\widehat{\mathbf{h}}^{{\mathrm{(1,2)}}}\in {\mathbb {C}}^{n(N_s-n)}$ be the vector defined as

5.17 $$\begin{aligned} \left( \widehat{\mathbf{h}}^{{\mathrm{(1,2)}}}\right) _{\alpha _{ij}}= {\widehat{h}}_{i,j}^{{\mathrm{(1,2)}}}~\text{ for }~ 1 \leqslant i \leqslant n ~\text{ and }~ 1 \leqslant j\leqslant N_s-n. \end{aligned}$$

Define the Loewner matrix ${{\mathbb {L}}}^{(1,2)} \in {\mathbb {C}}^{n(N_s-n)\times n}$ with entries

5.18 $$\begin{aligned} \left( {{\mathbb {L}}}^{{{(1,2)}}}\right) _{\alpha _{ij}\ell } = \frac{{\widehat{h}}_{i,j}^{{\mathrm{(1,2)}}}-h_{i,\ell }}{{\widehat{{\xi }}}_j- {\xi }_\ell }, \end{aligned}$$

for $1 \leqslant i \leqslant n, \ 1 \leqslant j\leqslant N_s-n$, and $1 \leqslant \ell \leqslant n$. Then, using (5.18) and (5.17) in (5.16), we obtain

$$\begin{aligned} \sum _{i=1}^n\sum _{j=1}^{N_s-n} \left( \mathfrak {p}_2 ({\xi }_i,{\widehat{{\xi }}}_j) - {\widehat{h}}_{i,j}^{{\mathrm{(1,2)}}}\mathfrak {q}_2 ({\xi }_i,{\widehat{{\xi }}}_j)\right) ^2 = \Vert {{\mathbb {L}}}^{{{(1,2)}}}\mathbf{w}+ \widehat{\mathbf{h}}^{{\mathrm{(1,2)}}}\Vert _2^2, \end{aligned}$$

yielding the linearization of ${{\mathcal {J}}}_2$:

5.19 $$\begin{aligned} {{\mathcal {J}}}_2 \rightsquigarrow {\frac{1}{(N_s-n)n}} \left\| {{\mathbb {L}}}^{{{(1,2)}}}\mathbf{w}+ \widehat{\mathbf{h}}^{{\mathrm{(1,2)}}}\right\| _2^2. \end{aligned}$$

Using similar arguments and the explicit formula for $r_2({\widehat{{\xi }}}_j,{\xi }_i)$ from (4.23), the ${{\mathcal {J}}}_3$ term in (5.9) is linearized to

5.20 $$\begin{aligned} {{\mathcal {J}}}_3 \rightsquigarrow \frac{1}{(N_s-n)n} \left\| {{\mathbb {L}}}^{{{(2,1)}}}\mathbf{w}+ \widehat{\mathbf{h}}^{{\mathrm{(2,1)}}}\right\| _2^2, \end{aligned}$$

where the Loewner matrix ${{\mathbb {L}}}^{(2,1)} \in {\mathbb {C}}^{n(N_s-n)\times n}$ and the vector $\widehat{\mathbf{h}}^{{\mathrm{(2,1)}}}\in {\mathbb {C}}^{n(N_s-n)}$ are defined as

$$\begin{aligned} \left( {{\mathbb {L}}}^{{{(2,1)}}}\right) _{\gamma _{ji}k} = \frac{{\widehat{h}}_{j,i}^{{\mathrm{(2,1)}}}-h_{k,i}}{{\widehat{{\xi }}}_j- {\xi }_k} \quad \text{ and }\quad (\widehat{\mathbf{h}}^{{\mathrm{(2,1)}}})_{\gamma _{ji}} = {\widehat{h}}_{j,i}^{{\mathrm{(2,1)}}}, \end{aligned}$$

with $$1 \leqslant j \leqslant N_s-n, \ 1 \leqslant i\leqslant n$$, $$1 \leqslant k \leqslant n$$, and $$\gamma _{ji} = (j-1)n+i$$.

*Quadraticizing the* ${{\mathcal {J}}}_4$ *term* In this section we show how to relax the remaining term, ${{\mathcal {J}}}_4$, in the minimization problem (5.6). Note that this term includes $r_2({\widehat{{\xi }}}_i,{\widehat{{\xi }}}_j)$; i.e., $r_2(s,z)$ evaluated over ${\widehat{{\varvec{{\xi }}}}}\times {\widehat{{\varvec{{\xi }}}}}$. As we stated earlier, unlike $r_2({\xi }_i,{\widehat{{\xi }}}_j)$ ($r_2(s,z)$ over ${\varvec{{\xi }}}\times {\widehat{{\varvec{{\xi }}}}}$) or $r_2({\widehat{{\xi }}}_i,{\xi }_j)$ ($r_2(s,z)$ over ${\varvec{{\xi }}}\times {\widehat{{\varvec{{\xi }}}}}$), the numerator and denominator of the quantity $r_2({\widehat{{\xi }}}_i,{\widehat{{\xi }}}_j)$ is quadratic in the weights $w_\ell$. Therefore, relaxing the (*ij*)th term in ${{\mathcal {J}}}_4$ via multiplying it out with its denominator, will not yield a linear term, but rather a quadratic one. Therefore, even the relaxed problem cannot be solved as a linear LS problem. This is what we establish next.

Similar to (5.15), relax the (*ij*)th term in (5.10) using

5.21 $$\begin{aligned} r_2({\widehat{{\xi }}}_i,{\widehat{{\xi }}}_j) - {\widehat{h}}_{i,j}^{{\mathrm{(2,2)}}}&= \dfrac{1}{\mathfrak {q}_2({\widehat{{\xi }}}_i,{\widehat{{\xi }}}_j)} \left( \mathfrak {p}_2({\widehat{{\xi }}}_i,{\widehat{{\xi }}}_j) - {\widehat{h}}_{i,j}^{{\mathrm{(2,2)}}}\mathfrak {q}_2 ({\widehat{{\xi }}}_i,{\widehat{{\xi }}}_j) \right) \nonumber \\&\rightsquigarrow \mathfrak {p}_2 ({\widehat{{\xi }}}_i,{\widehat{{\xi }}}_j)- {\widehat{h}}_{i,j}^{{\mathrm{(2,2)}}}\mathfrak {q}_2 ({\widehat{{\xi }}}_i,{\widehat{{\xi }}}_j). \end{aligned}$$

Using (4.21), we obtain

5.22 $$\begin{aligned} r_2({\widehat{{\xi }}}_i,{\widehat{{\xi }}}_j) = = \frac{ \mathfrak {p}_2 ({\widehat{{\xi }}}_i,{\widehat{{\xi }}}_j)}{\mathfrak {q}_2 ({\widehat{{\xi }}}_i,{\widehat{{\xi }}}_j)} = \frac{ \sum _{k=1}^n \sum _{\ell =1}^n \frac{ w_k w_\ell h_{k,\ell } }{({\widehat{{\xi }}}_i - {\xi }_k)({\widehat{{\xi }}}_j - {\xi }_\ell ) }}{(1+ \sum _{k=1}^n \frac{w_k }{{\widehat{{\xi }}}_i- {\xi }_k})(1+ \sum _{\ell =1}^n \frac{w_\ell }{{\widehat{{\xi }}}_j- {\xi }_\ell })} . \end{aligned}$$

Inserting $\mathfrak {p}_2 ({\widehat{{\xi }}}_i,{\widehat{{\xi }}}_j)$ and $\mathfrak {q}_2 ({\widehat{{\xi }}}_i,{\widehat{{\xi }}}_j)$ from (5.22) into (5.21) and re-arranging the terms yield

5.23 $$\begin{aligned}&\mathfrak {p}_2 ({\widehat{{\xi }}}_i,{\widehat{{\xi }}}_j)- {\widehat{h}}_{i,j}^{{\mathrm{(1,2)}}}\mathfrak {q}_2 ({\xi }_i,{\widehat{{\xi }}}_j) \nonumber \\&\quad = -\left( \sum _{k=1}^n \sum _{\ell =1}^n \frac{w_k w_\ell ({\widehat{h}}_{i,j}^{{\mathrm{(2,2)}}}-h_{k,\ell })}{({\widehat{{\xi }}}_i- {\xi }_k)({\widehat{{\xi }}}_j- {\xi }_\ell )} + \sum _{k=1}^n \frac{w_k {\widehat{h}}_{i,j}^{{\mathrm{(2,2)}}}}{{\widehat{{\xi }}}_i- {\xi }_k} + \sum _{\ell =1}^n \frac{w_\ell {\widehat{h}}_{i,j}^{{\mathrm{(2,2)}}}}{{\widehat{{\xi }}}_j- {\xi }_\ell } - {\widehat{h}}_{i,j}^{{\mathrm{(2,2)}}}\right) . \end{aligned}$$

Note that the expression in (5.23) is quadratic in $w_k$, as anticipated.

As we did for ${{\mathcal {J}}}_1$, ${{\mathcal {J}}}_2$ and ${{\mathcal {J}}}_3$, to express the resulting expression more compactly in matrix form, we introduce the (2D) Loewner matrix ${{\mathbb {L}}}^{{\mathrm{(2,2)}}}\in {\mathbb {C}}^{(N_s-n)^2 \times n^2}$ as

5.24 $$\begin{aligned} ({{\mathbb {L}}}^{{\mathrm{(2,2)}}})_{\alpha _{ij} \beta _{k\ell }} = \frac{{\widehat{h}}_{i,j}^{{\mathrm{(2,2)}}}-h_{k,\ell }}{({\widehat{{\xi }}}_i- {\xi }_k)({\widehat{{\xi }}}_j - {\xi }_\ell )}, \end{aligned}$$

where $\alpha _{ij} = (i-1)(N_s-n)+j$ and $\beta _{k\ell } = (k-1)n+\ell$ with $i,j \in \{1,2,\ldots , N_s-n\}$ and $k,\ell \in \{1,2,\ldots ,n\}$. Then, the $\alpha _{ij}$th entry of the vector ${{\mathbb {L}}}^{{\mathrm{(2,2)}}}(\mathbf{w}\otimes \mathbf{w}) \in {\mathbb {C}}^{(N_s-n)^2}$ is

5.25 $$\begin{aligned} \Big ( {{\mathbb {L}}}^{{\mathrm{(2,2)}}}(\mathbf{w}\otimes \mathbf{w}) \Big )_ {\alpha _{ij}} = -\sum _{k=1}^n \sum _{\ell =1}^n \frac{w_k w_\ell (h_{k,\ell }-{\widehat{h}}_{i,j}^{{\mathrm{(2,2)}}})}{({\widehat{{\xi }}}_i- {\xi }_k)({\widehat{{\xi }}}_j- {\xi }_\ell )}, \end{aligned}$$

thus recovering the first sum in (5.23). Next, introduce the matrices $\mathbf{U}_1, \mathbf{U}_2 \in {\mathbb {C}}^{(N_s-n)^2 \times n}$ such that for $1\leqslant k,\ell \leqslant n$,

5.26 $$\begin{aligned} (\mathbf{U}_1)_{\alpha _{ij}k} = \frac{{\widehat{h}}_{i,j}^{{\mathrm{(2,2)}}}}{{\widehat{{\xi }}}_i- {\xi }_k} \quad \text{ and }\quad (\mathbf{U}_2)_{\alpha _{ij}\ell } = \frac{{\widehat{h}}_{i,j}^{{\mathrm{(2,2)}}}}{{\widehat{{\xi }}}_j- {\xi }_\ell }. \end{aligned}$$

Using $\mathbf{U}_1$ and $\mathbf{U}_2$ in (5.26), the last two sums in (5.23) are compactly written as

5.27 $$\begin{aligned} \sum _{k=1}^n \frac{w_k {\widehat{h}}_{i,j}^{{\mathrm{(2,2)}}}}{{\widehat{{\xi }}}_i- {\xi }_k} = \mathbf{U}_1 \mathbf{w}\quad \text{ and }\quad \sum _{\ell =1}^n \frac{w_\ell {\widehat{h}}_{i,j}^{{\mathrm{(2,2)}}}}{{\widehat{{\xi }}}_j- {\xi }_\ell } = \mathbf{U}_2 \mathbf{w}. \end{aligned}$$

Define $\mathbf{U}= \mathbf{U}_1+\mathbf{U}_2$. Then using (5.26), we write

5.28 $$\begin{aligned} (\mathbf{U})_{\alpha _{ij}k} =(\mathbf{U}_1)_{\alpha _{ij}k} + (\mathbf{U}_2)_{\alpha _{ij}k} = \frac{{\widehat{h}}_{i,j}^{{\mathrm{(2,2)}}}({\widehat{{\xi }}}_i+{\widehat{{\xi }}}_j-2{\xi }_k)}{({\widehat{{\xi }}}_i- {\xi }_k)({\widehat{{\xi }}}_j- {\xi }_k)}. \end{aligned}$$

Insert (5.24) and (5.28) into (5.22) to obtain

5.29 $$\begin{aligned} \sum _{i=1}^{N_s-n} \sum _{j=1}^{N_s-n} (\mathfrak {p}_2 ({\widehat{{\xi }}}_i,{\widehat{{\xi }}}_j) - {\widehat{h}}_{i,j}^{{\mathrm{(2,2)}}}\mathfrak {q}_2 ({\xi }_i,{\widehat{{\xi }}}_j))^2 = \left\| {{\mathbb {L}}}^{{\mathrm{(2,2)}}}( \mathbf{w}\otimes \mathbf{w}) + \mathbf{U}\mathbf{w}+ \widehat{\mathbf{h}}^{{\mathrm{(2,2)}}}\right\| _2^2, \end{aligned}$$

where $\widehat{\mathbf{h}}^{{\mathrm{(2,2)}}}\in {\mathbb {C}}^{(N_s-n)^2}$ is the vector defined as

5.30 $$\begin{aligned} (\widehat{\mathbf{h}}^{{\mathrm{(2,2)}}})_{\alpha _{ij}} = {\widehat{h}}_{i,j}^{{\mathrm{(2,2)}}}, \end{aligned}$$

with $\alpha _{ij} = (i-1)(N_s-n)+j$ as before and $1 \leqslant i,j\leqslant N_s-n$. The expression (5.29) yields the final relaxation of ${{\mathcal {J}}}_4$:

5.31 $$\begin{aligned} {{\mathcal {J}}}_4 \rightsquigarrow {\frac{1}{(N_s-n)^2}} \left\| {{\mathbb {L}}}^{{\mathrm{(2,2)}}}( \mathbf{w}\otimes \mathbf{w}) + \mathbf{U}\mathbf{w}+ \widehat{\mathbf{h}}^{{\mathrm{(2,2)}}}\right\| _2^2 . \end{aligned}$$

#### Solving the LS Problem in Step *n*

Now we are ready to describe the resulting LS problem to solve in Step *n* of AAA-LQO. Combining the relaxations ${{\mathcal {J}}}_1$, ${{\mathcal {J}}}_2$, ${{\mathcal {J}}}_3$, and ${{\mathcal {J}}}_4$ as given in (5.14), (5.19), (5.20), and (5.31), in the *n*th step of the algorithm, we need to solve the quadraticized minimization problem

5.32 $$\begin{aligned}&\min \limits _{\mathbf{w}} \left\{ \rho _1 \left\| {{\mathbb {L}}}\mathbf{w}+ {\widehat{\mathbf{h}}}\right\| _2^2 \right. + \rho _2\left( \left\| {{\mathbb {L}}}^{{{(1,2)}}}\mathbf{w}+ \widehat{\mathbf{h}}^{{\mathrm{(1,2)}}}\right\| _2^2 + \left\| {{\mathbb {L}}}^{{{(2,1)}}}\mathbf{w}+ \widehat{\mathbf{h}}^{{\mathrm{(2,1)}}}\right\| _2^2 \right) \nonumber \\&\qquad +\left. \rho _3 \left\| {{\mathbb {L}}}^{{\mathrm{(2,2)}}}( \mathbf{w}\otimes \mathbf{w}) + \mathbf{U}\mathbf{w}+ \widehat{\mathbf{h}}^{{\mathrm{(2,2)}}}\right\| _2^2 \right\} , \end{aligned}$$

where

5.33 $$\begin{aligned} \rho _1 = \frac{1}{N_s-n},~\rho _2 =\frac{1}{(N_s-n)n},~\text{ and }~\rho _3= \frac{1}{(N_s-n)^2}. \end{aligned}$$

Note that due to the last term, the optimization problem (5.32) is no longer a linear LS problem, nevertheless can be solved efficiently. One can explicitly compute the gradient (and Hessian) of the cost function and can apply a well-established (quasi)-Newton formulation [33]. If we were to have a one-step algorithm whose solution were determined by (5.32), we would employ these techniques. However, solving (5.32) is only one step of our proposed iterative algorithm. As the iteration proceeds (and as *n* increases), the vector $\mathbf{w}$ (and the data-partition) will be updated and the new optimization problem with a larger-dimension needs to be solved. Therefore, we will approximately solve (5.32) in every step.

One can obtain an approximate solution to (5.32) in various ways. In our formulation, we will first solve part of the problem (5.32) that can be written as a linear least-squares problem in $\mathbf{w}$, namely

5.34 $$\begin{aligned} \min \limits _{\mathbf{w}} \left\{ \rho _1 \left\| {{\mathbb {L}}}\mathbf{w}+ {\widehat{\mathbf{h}}}\right\| _2^2 + \rho _2\left( \left\| {{\mathbb {L}}}^{{{(1,2)}}}\mathbf{w}+ \widehat{\mathbf{h}}^{{\mathrm{(1,2)}}}\right\| _2^2 + \left\| {{\mathbb {L}}}^{{{(2,1)}}}\mathbf{w}+ \widehat{\mathbf{h}}^{{\mathrm{(2,1)}}}\right\| _2^2 \right) \right\} . \end{aligned}$$

The optimization problem (5.34) is a classical linear least-squares problem:

5.35 $$\begin{aligned} {\tilde{\mathbf{w}}} = {\displaystyle {\underset{\mathbf{w}}{{\text {arg\,min}} }}} \left\| ~ \left[ \begin{array}{l} \rho _1 {{\mathbb {L}}}\\ \rho _2{{\mathbb {L}}}^{{{(1,2)}}}\\ \rho _2{{\mathbb {L}}}^{{{(2,1)}}}\end{array} \right] \mathbf{w}+ \left[ \begin{array}{l} {\widehat{\mathbf{h}}}\\ \widehat{\mathbf{h}}^{{\mathrm{(1,2)}}}\\ \widehat{\mathbf{h}}^{{\mathrm{(2,1)}}}\end{array} \right] ~ \right\| _2. \end{aligned}$$

With ${\tilde{\mathbf{w}}}$, we further relax the last term in (5.32) as

5.36 $$\begin{aligned} \rho _3 \Vert {{\mathbb {L}}}^{{\mathrm{(2,2)}}}( {\mathbf{w}} \otimes \mathbf{w}) + \mathbf{U}\mathbf{w}+ \widehat{\mathbf{h}}^{{\mathrm{(2,2)}}}\Vert _2^2 \rightsquigarrow \rho _3 \Vert {{\mathbb {L}}}^{{\mathrm{(2,2)}}}( {\tilde{\mathbf{w}}} \otimes \mathbf{w}) + \mathbf{U}\mathbf{w}+ \widehat{\mathbf{h}}^{{\mathrm{(2,2)}}}\Vert _2^2. \end{aligned}$$

Using the equality ${{\mathbb {L}}}^{{\mathrm{(2,2)}}}( {\tilde{\mathbf{w}}} \otimes \mathbf{w}) = {{\mathbb {L}}}^{{\mathrm{(2,2)}}}( {\tilde{\mathbf{w}}} \otimes {\hat{\mathbf{I}}}_n) \mathbf{w}$, we rewrite (5.36) as

5.37 $$\begin{aligned}  \rho _3 \Vert {{\mathbb {L}}}^{{\mathrm{(2,2)}}}( {\tilde{\mathbf{w}}} \otimes \mathbf{w}) + \mathbf{U}\mathbf{w}+ \widehat{\mathbf{h}}^{{\mathrm{(2,2)}}}\Vert _2^2 = \rho _3 \Vert {{\mathbb {T}}}\mathbf{w}+ \widehat{\mathbf{h}}^{{\mathrm{(2,2)}}}\Vert _2^2, \end{aligned}$$

where the matrix ${{\mathbb {T}}}\in {\mathbb {C}}^{(N_s-n)^2 \times n}$ is given by

5.38 $$\begin{aligned} {{\mathbb {T}}}= {{\mathbb {L}}}^{{\mathrm{(2,2)}}}( {\tilde{\mathbf{w}}} \otimes \mathbf{I}) + \mathbf{U}. \end{aligned}$$

Then, finally using (5.37) in place of the last term in (5.32), we obtain a minimization problem that is now a linear LS problem. Thus, the solution to our final approximation to (5.32) is given by

5.39 $$\begin{aligned} {\mathbf{w}}_{\star } = {\displaystyle {\underset{\mathbf{w}}{{\text {arg\,min}} }}} \left\| ~\left[ \begin{array}{l} \rho _1{{\mathbb {L}}}\\ \rho _2 {{\mathbb {L}}}^{{{(1,2)}}}\\ \rho _2 {{\mathbb {L}}}^{{{(2,1)}}}\\ \rho _3 {{\mathbb {T}}}\end{array} \right] \mathbf{w}+\left[ \begin{array}{l} {\widehat{\mathbf{h}}}\\ \widehat{\mathbf{h}}^{{\mathrm{(1,2)}}}\\ \widehat{\mathbf{h}}^{{\mathrm{(2,1)}}}\\ \widehat{\mathbf{h}}^{{\mathrm{(2,2)}}}\end{array} \right] ~ \right\| _2. \end{aligned}$$

Therefore, in the *n*th step of AAA-LQO, the optimization problem (5.6) is relaxed and the solution of this relaxed problem (the weights) is given by (5.39). The algorithms proceeds with the updated weights as we discuss next. We also note that the second relaxation approach in (5.31) can be replaced by a few steps of a (quasi)-Newton method.

### Partition Update via the Greedy Selection

Given the partition (5.1) in the Step *n* of the algorithm, Sect. 5.1 showed how to choose the barycentric weights $\mathbf{w}$ to minimize a joint LS measure over the uninterpolated data set. The only remaining component of the proposed approach is, then, to choose the next support point $\xi _{n+1}$ and to update the data partition (5.1) (so that we repeat Sect. 5.1 for the updated partition until a desired tolerance achieved.) In other words, we will move one sampling point from the LS set ${\widehat{{\varvec{{\xi }}}}}$ to the interpolation set ${\varvec{{\xi }}}$. Which point to move from ${\widehat{{\varvec{{\xi }}}}}$ to ${\varvec{{\xi }}}$ will be done in a greedy manner. To re-emphasize the iterative nature of the overall algorithm, at this Step *n* of the algorithm, we will denote by $r_1^{(n)}(s)$ and $r_2^{(n)}(s,z)$ the two transfer functions of the current LQO approximant. (Note that we dropped the superscripts in Sect. 5.1 to simplify the notation there.)

We start by defining two constants based on the data:

5.40 $$\begin{aligned} M_1&= \max \limits _{s \in \Omega } \vert H_1(s) \vert , \quad M_2 = \max \limits _{s\in \Omega , z \in \Omega } \vert H_2(s,z) \vert , \end{aligned}$$

where $\Omega$ denotes the full sampling set $\Omega = \{s_1,s_2,\ldots ,s_{N_s}\}$. For the current approximant in Step *n*, introduce the two absolute error measures, namely deviations in the linear and quadratic parts:

5.41 $$\begin{aligned} \epsilon _1^{(n)} = \max \limits _{s \in \Omega } \vert H_1(s) - r_1^{(n)}(s) \vert \quad \text{ and }\quad \epsilon _2^{(n)} = \max \limits _{s,z \in \Omega } \vert H_2(s,z) - r_2^{(n)}(s,z) \vert . \end{aligned}$$

The next support point ${\xi }_{n+1}$ is chosen by means of a greedy search over the set $\Omega \setminus \{{\xi }_{1},\ldots ,{\xi }_n\}$ using the error measures $\epsilon _1^{(n)}$ and $\epsilon _2^{(n)}$. More precisely, if $\epsilon _1^{(n)}/N>\epsilon _2^{(n)}/N^2$, then ${\xi }_{n+1} = {\displaystyle {\underset{s\in \Omega }{{\text {arg\,max}} }}\,\vert H_1(s) - r_1^{(n)}(s) \vert }$. On the other hand, if $\epsilon _1^{(n)}/N<\epsilon _2^{(n)}/N^2$, define $s^{(n+1)}$ and $z^{(n+1)}$ using

$$\begin{aligned} (s^{(n+1)},z^{(n+1)})= {\displaystyle {\underset{s,z\in \Omega }{{\text {arg\,max}} }}\,\vert H_2(s,z) - r_2(s,z)} \vert . \end{aligned}$$

Now the question is whether to choose either $s^{(n+1)}$ or $z^{(n+1)}$ as ${\xi }_{n+1}$. If only one of them was already a support point, then we choose the other one as ${\xi }_{n+1}$. If neither $s^{(n+1)}$ nor $z^{(n+1)}$ was previously chosen as a support point, then we compare $\vert H_1(s^{(n+1)}) - r_1^{(n)}(s^{(n+1)}) \vert$ and $\vert H_1(z^{(n+1)}) - r_1^{(n)}(z^{(n+1)}) \vert$, and choose ${\xi }_{n+1}$ as the one that yields the higher deviation in the first transfer function. Clearly, both cannot be already a support point due to the interpolation property.

#### Remark 5.1

Instead of considering the full grid of pairs of sampling points (*s*, *z*) and the associated measurements, we could consider a sparser grid for $H_2(s,z)$ samples. More precisely, instead of using the full grid in (4.2) that contains $N_s^2$ pairs, we could use the sample set $\{H_2(s_i,s_j)\} \in {\mathbb {C}}~~\text{ where }~~s_i,s_j \in {\mathbb {C}}$ with $i \in {{\mathcal {I}}} , \ j \in {{\mathcal {J}}}$, ${{\mathcal {I}}} , {{\mathcal {J}}} \subset {{\mathbb {Z}}}_{+}$ with cardinalities satisfying $\vert {{\mathcal {I}}} \vert = N_s^{( {{\mathcal {I}}} )} < N_s$, and $\vert {{\mathcal {J}}} \vert = N_s^{( {{\mathcal {J}}} )} < N_s$. This can be viewed as a sub-sampling approach for reducing the complexity of the LS problem by reducing the number of measurements from $N_s^2$ to $N_s^{( {{\mathcal {I}}} )} N_s^{( {{\mathcal {J}}} )}$ for the second transfer function. This sparse sampling will reduce the dimension of the Loewner matrix ${{\mathbb {L}}}^{{\mathrm{(2,2)}}}\in {\mathbb {C}}^{(N_s-n)^2 \times n^2}$ in Sect. 5.1.1 by reducing the number of rows from $(N_s-n)^2$ to $(N_s^{( {{\mathcal {I}}} )}-n)(N_s^{( {{\mathcal {J}}} )}-n)$. However, this modification would require changing the greedy selection scheme accordingly to make sure that all possible combinations of selected points appear in the sparser grid. We skip this aspect in our examples and work with the full data set.

### The Proposed Algorithm: AAA-LQO

Now, we have all the pieces to describe the algorithmic framework for the proposed method AAA-LQO, the AAA algorithm for LQO systems.

Given the full LQO data (4.1) and (4.2), we initiate the approximant ($n=0$) by choosing $r_1^{(0)}(s)$ as the average of $H_1(s)$ samples and $r_2^{(0)}(s,z)$ as the average of $H_2(s,z)$ samples. Then, using the greedy selection strategy of Sect. 5.2 we update the partition (5.1) and solve for the barycentric weights as in Sect. 5.1, more specifically by solving (5.39). Let $n_{\mathrm{max}}$ denote the largest dimension permitted for the data-driven LQO system $\widehat{\Sigma }_{\textsf {LQO}}$ and and let $\epsilon$ denote the relative error tolerance. Then, AAA-LQO terminates either when the prescribed dimension $n_{\mathrm{max}}$ is reached, or when the prescribed error tolerance is achieved, namely

5.42 $$\begin{aligned} \max ( \epsilon _1^{(n)}/M_1, \epsilon _2^{(n)}/M_2) < \tau . \end{aligned}$$

A sketch of AAA-LQO is given in Algorithm 1. 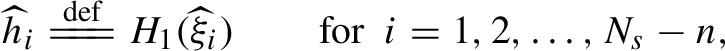


#### Remark 5.2

Note that, by choosing complex-conjugate sampling points and sampled values, one can enforce the fitted models to be real-valued. This means that if a particular point ${\xi }_i$ is selected, its conjugate is also automatically selected (${\xi }_{i+1} = {\bar{{\xi }}}_i$), hence increasing the degree of the fitted functions. This is performed in both examples presented in Sect. 6.

## Numerical Examples

We test AAA-LQO, as given in Algorithm 1, on two LQO systems. We also apply the original AAA algorithm (from the linear case) to the data corresponding to the first (linear) transfer function only. Therefore, we construct two approximants: (1) A data-driven LQO approximant using AAA-LQO and (2) A data-driven linear approximant using AAA. Both approximants are real-valued, enforced by using a data set that is closed under complex conjugation.

### Example 1

First, we use a single-input/single-output version of the ISS 1R Model from the SLICOT MOR benchmark collection [14]. We construct an LQO system from this linear model by adding a quadratic output with the choice of $\mathbf{M}= 0.6 \mathbf{I}_{270}+ 0.3\mathbf{I}^{(-1)}_{270}+0.3\mathbf{I}^{(+1)}_{270} \in {\mathbb {R}}^{270 \times 270}$, which scales the product of the state variable with itself in the output equation. Here, $\mathbf{I}^{(k)}_{270}$ denotes a quasi-diagonal matrix for which the entries of ones are shifted from the main diagonal based on the integer *k* ($k>0$ stands for shifting upward, while $k<0$ is used for shifting downward - also, note that $\mathbf{I}^{(0)}_{270}= \mathbf{I}_{270}$).

**Fig. 1:**
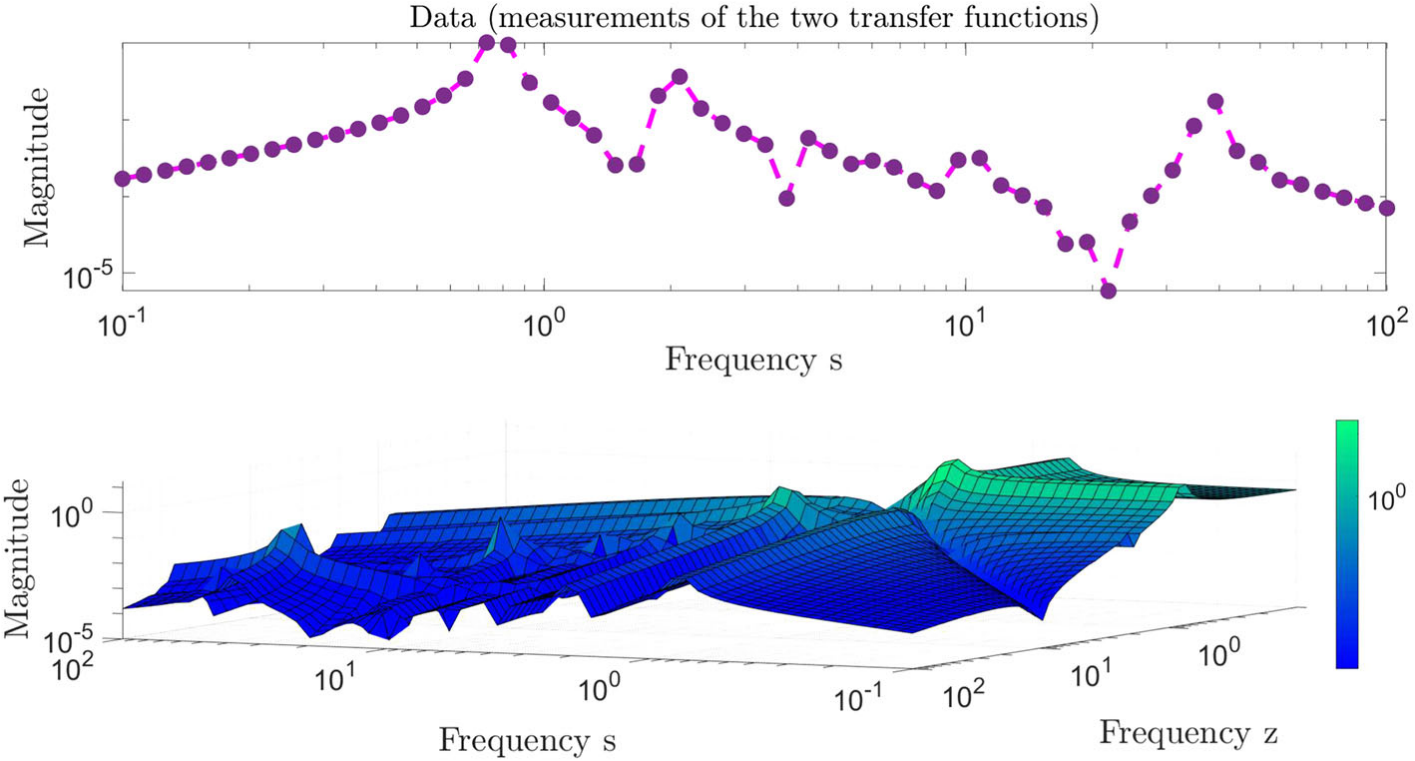
Measurements of the two transfer functions; $H_1(s)$ (top) and $H_2(s,z)$ (bottom)

We collect the following data: pick 60 logarithmically-spaced points in the interval $[10^{-1},10^2] \mathrm{i}$ and add its conjugate pairs in $[-10^{2},-10^{1})] \mathrm{i}$ to have $N_s=120$ sampling points $\{s_i\}$ and the samples $\{H_1(s_i)\}$ for $i=1,2,\ldots ,N_s$ as in (4.1). Then, as in (4.2), we sample the second-transfer function at $H_2(s_i,s_j)$ for $i,j=1,2,\ldots ,N_s$. The sampled data are depicted in Fig. 1, where we display the measurements evaluated only on the “positive side” of the imaginary axis and skip plotting the conjugate data.

We apply Algorithm 1 with $n_{\mathrm{max}}=30$ and $\tau = 10^{-2}$ (relative tolerance value corresponding to $99\%$ approximation error on the data). With these variables, AAA-LQO yields a data-driven LQO model of order $n=18$.

Using only the $\{ H_1(s_i)\}$ samples (corresponding to the linear observation map), we apply AAA and obtain a data-driven linear approximant of order $n=18$. The AAA approximant is constructed to simply illustrate that a linear dynamical system approximation is not sufficient to accurately represent the underlying LQO system.

**Fig. 2:**
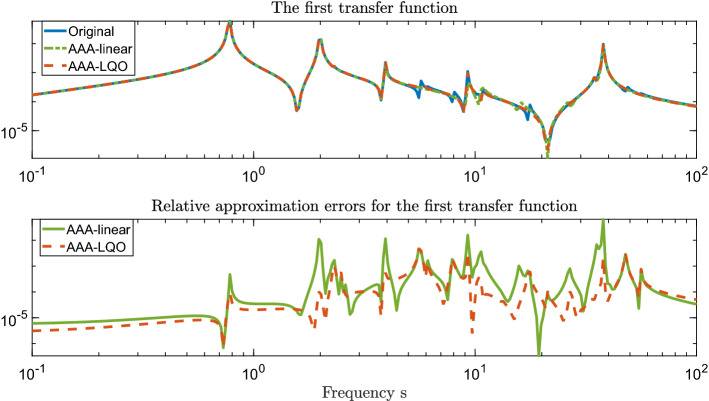
First transfer function approximation

In the top plot of Fig. 2, we show the magnitude of the first transfer function $H_1(s)$ of the original system together with that of the linear AAA model and the first transfer function ($r_1^{(n)}(s)$) of the AAA-LQO model. As expected, AAA model does a good job in matching the linear part of the output. Similarly, the AAA-LQO model also matches $H_1(s)$ accurately. To better illustrate this, in the bottom plot of Fig. 2, we depict the magnitude of the approximation errors in $H_1(s)$. The plot reveals that for this specific choice of $\tau$, the AAA-LQO model has a smaller error for most of the frequency values, even in approximating $H_1(s)$. This happens despite the fact that it focuses on both $H_1(s)$ and $H_2(s,z)$ unlike the AAA model, which only tries to approximate $H_1(s)$. However, we do not claim this to be the general case. We have observed that for some lower values of $\tau$, e.g., $\tau = 10^{-4}$, AAA model has outperformed AAA-LQO model in approximating $H_1(s)$ (as one would expect) even though the AAA-LQO model has still provided a high-fidelity approximation to $H_1(s)$.

**Fig. 3:**
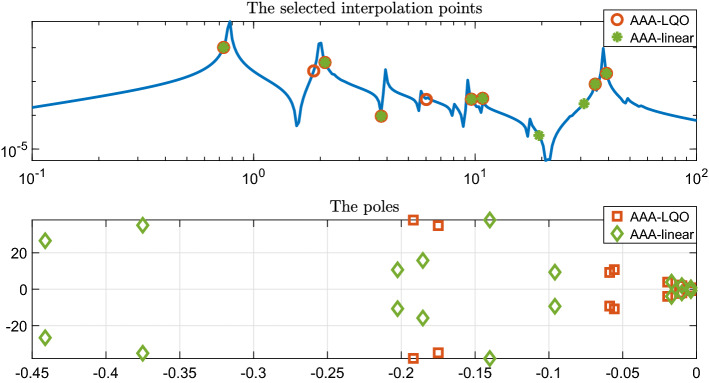
Support points (top) and poles (bottom) for the two AAA reduced-order models

In Fig. 3 we depict the selected support points (interpolation points) for both AAA and AAA-LQO algorithms (without the complex conjugate pairs), as well as the poles of the learned models (i.e., the eigenvalues of ${{\hat{\mathbf{A}}}}$ in both cases). Note that there are 9 complex conjugate pairs of support points for each method. Even though some of the support points of AAA and AAA-LQO overlap, two of the pairs are different. This difference causes a big deviation in the the pole pattern as shown in the bottom plot, illustrating that even the linear part of the AAA-LQO approximant, i.e., $r_1^{(n)}(s)$, is fundamentally different than the linear AAA model. This is expected since AAA-LQO constructs $r_1^{(n)}(s)$ and $r_2^{(n)}(s,z)$ together by minimizing a joint LS measure in both $H_1(s)$ and $H_2(s,z)$.

**Fig. 4:**
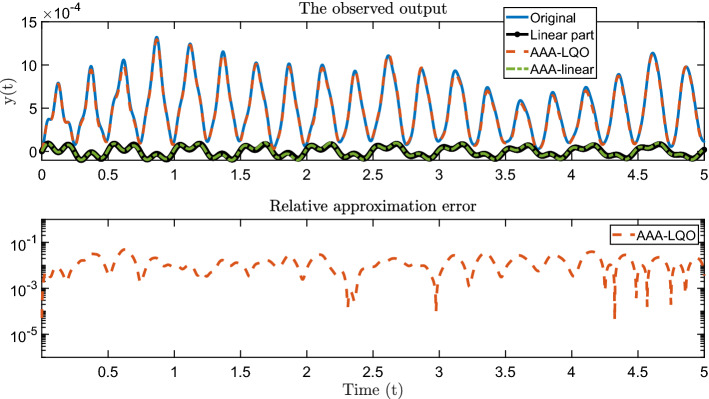
Time-domain simulations: output of the original and data-driven models (top) and approximation error (bottom)

To show the overall performance of AAA-LQO in accurately approximating not only $H_1(s)$ but also $H_2(s,z)$ (the full LQO behavior), we perform a time-domain simulation of the original LQO system $\Sigma _{\textsf {LQO}}$, the data-driven AAA-LQO model $\widehat{\Sigma }_{\textsf {LQO}}$, and the linear AAA model by using $u(t) = 0.5 \cos (4 \pi t)$ as the control input. During the simulation of the original system $\Sigma _{\textsf {LQO}}$, we also compute only the linear part of the output, which the AAA model should approximate well. The results are given in the top plot of Fig. 4. The first observation is that the output of $\widehat{\Sigma }_{\textsf {LQO}}$ from AAA-LQO accurately replicates the output of $\Sigma _{\textsf {LQO}}$. On the other hand, the linear AAA model completely misses the quadratic output and is only able to approximate the linear component in the output, as expected. The approximation error in the output corresponding to $\widehat{\Sigma }_{\textsf {LQO}}$ is depicted in the bottom plot of Fig. 4.

**Fig. 5:**
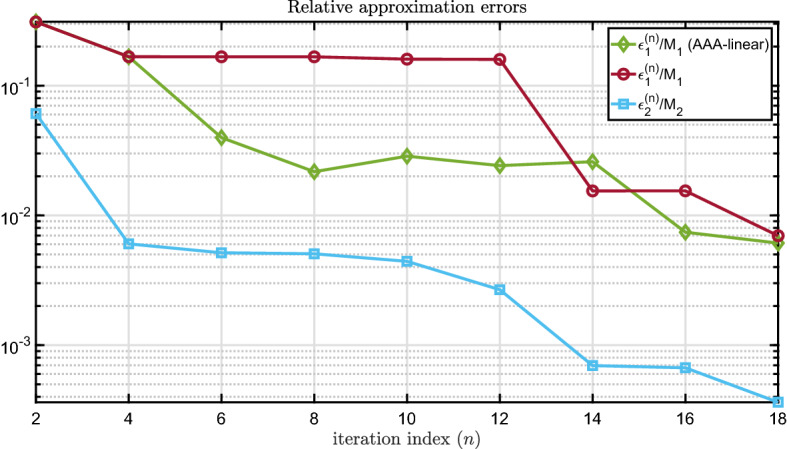
Relative approximation errors in each step

**Fig. 6:**
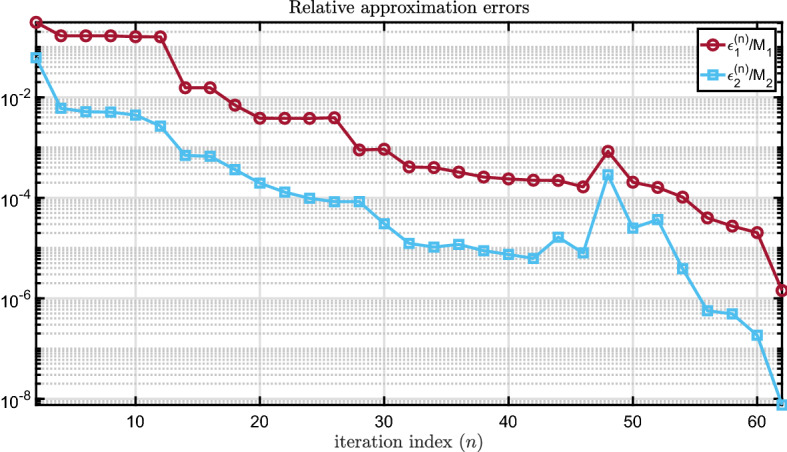
Relative approximation errors in each step

In Fig. 5 we show the convergence behavior of AAA-LQO by plotting the evolution of the relative approximation errors ($\epsilon _1^{(n)}/M_1$ and $\epsilon _2^{(n)}/M_2)$ for all even values of *n*. The figure illustrates that after $n=18$, both relative errors fall below the given tolerance of $10^{-2}$ and the algorithm terminates. For reference, we also depict the convergence behavior of AAA on the same figure. The stagnancy of the $\epsilon _1^{(n)}/M_1$ error curve from $n=2$ to $n=12$ results from the fact that during those steps the greedy selection was based on the $\epsilon _2^{(n)}$ term, which was the dominant *absolute value* error term. One can observe that during these steps, $\epsilon _2^{(n)}/M_2$ continues to decay slightly. A more detailed illustration is given in Fig. 6, where the *n* is varied from 2 to 62.

To investigate how the order of the AAA-LQO model varies based on the stopping tolerance, we set $n_{\mathrm{max}} = 100$ and run AAA-LQO for four tolerance values $\tau = 10^{-2}$, $\tau = 10^{-3}$, $\tau = 10^{-4}$, and $\tau = 10^{-5}$. The results are displayed in Table  1. For the case of $\tau = 10^{-5}$, in Fig. 6 we depict the convergence behavior of AAA-LQO by plotting $\epsilon _1^{(n)}/M_1$ and $\epsilon _2^{(n)}/M_2$ during the iteration.

### Example 2

This model taken from [35] corresponds to an LQO system whose output measures a variance in the state-variable. A linear mass-spring-damper SISO dynamical system was modified in [34] by means of stochastic modeling, by replacing the physical parameters by independent random variables, yielding a linear dynamical system with multiple outputs. Based on this multiple output system, a SISO LQO system was derived in [35] where the output corresponds to the variance of the original output (and thus is quadratic in nature). We refer the reader to [35] for further details. We obtain the measurements from a version of this model corresponding to an underlying LQO system of order ${{\mathcal {N}}} = 960$.

The main difference from the previous example is that, in this model the observed output does not have a linear component and depends on the state variable solely quadratically, i.e., $\mathbf{c}= {{\mathbf {0}}}$ in (2.1). This means that $H_1(s) = 0$.

**Fig. 7:**
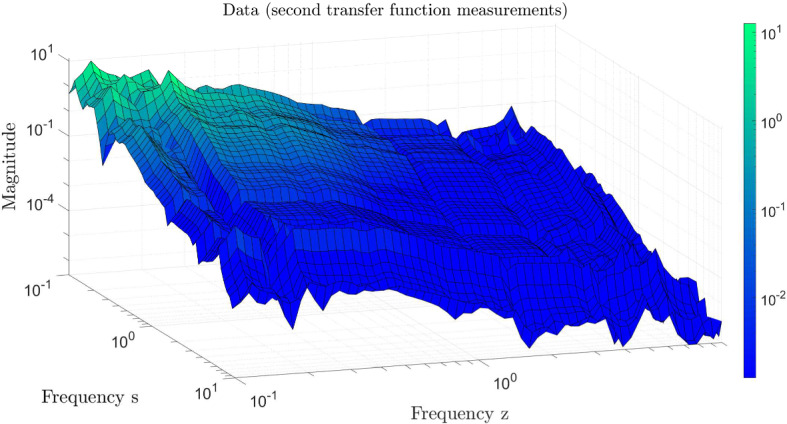
Measurements of the second transfer function

As the sampling points $\{s_i\}$, we choose 60 logarithmically spaced points over the interval $[10^{-1},10^1]\mathrm{i}$ together with its conjugate pairs, leading to $N_s = 120$ samples. Since $H_1(s) =0$, we only need to sample $H_2(s_i,s_j)$ for $i,j=1,2,\ldots ,N_s$. The corresponding data for the second transfer function are depicted in Fig. 7.

**Table 1:** Tolerance values $\tau$ versus the order *n*

$$\tau $$	$$ 10^{-2}$$	$$ 10^{-3}$$	$$ 10^{-4}$$	$$ 10^{-5}$$
*n*	18	28	56	62

**Fig. 8:**
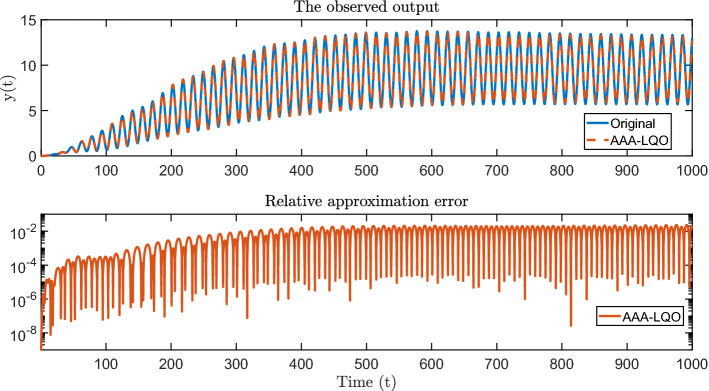
Time-domain simulations; output of the original and the reduced system (up) + approximation error (down)

We apply AAA-LQO with $n_{\mathrm{max}}=50$ and $\tau = 10^{-3}$ (relative stopping criterion), obtaining an LQO model of order $n=30$. To show the accuracy of the approximant, we perform time-domain simulations of the full model and the approximant with the input $u(t) = \sin (0.2 t)$. We depict the observed outputs in the top plot of Fig. 8, illustrating an accurate approximation. The corresponding output error is plotted in the bottom plot of Fig. 8.

**Fig. 9:**
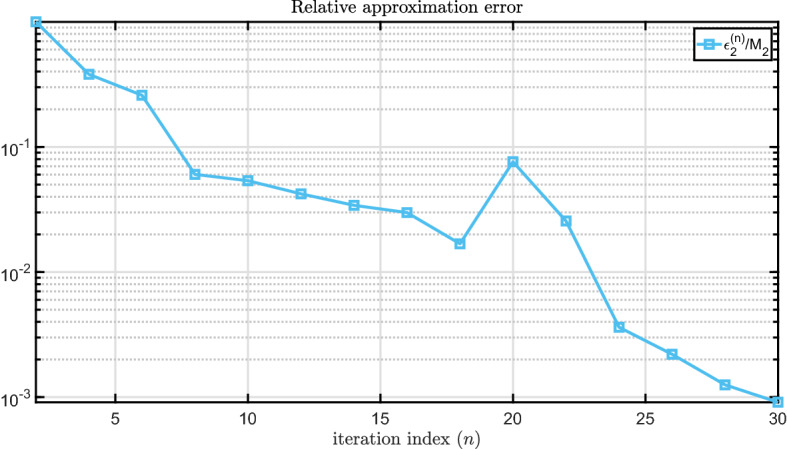
Maximum relative approximation error at each step

Finally, in Fig. 9 we show the convergence behavior of AAA-LQO by plotting the evolution of relative approximation error $\epsilon _2^{(n)}/M_2$.

#### Remark 6.1

Since AAA-LQO uses a greedy selection scheme and is not a descent algorithm, there is no theoretical guarantee that the maximum approximation error will decrease monotonically. This can be seen in Figs. 5, 6 and 9. This behavior was also observed in the original AAA algorithm; see, e.g, Application 6.3 in [32]. However, numerically the error indeed decreases monotonically with *n* in most cases.

#### The Case of Noisy Data

In practice, the frequency-domain data to be used in rational approximation algorithms are often corrupted by noise. The recent works [15, 20, 21] have studied the effects of noisy data on some of the frequency-domain based rational approximation for linear dynamics with linear output, such as the AAA [32], Loewner [31], and RKFIT [10], and Vector Fitting (VF) [22] frameworks. It was illustrated in [20, 21] that the methods such as RKFIT, VF and AAA with a (partial) least-squares formulation are more robust to noise in the measurements compared to the purely interpolatory Loewner framework.

In what follows, using the model in Example 2 we present a simple numerical test-case to study the effect of noise on AAA-LQO. We will artificially corrupt the measurements of the second transfer function with uniformly distributed numbers in the interval $(0,\zeta )$, where $\zeta$ is the “noise level”. We will be using moderate noise levels, i.e., $\zeta < 10^{-3} M_2$, with $M_2$ defined as in (5.40). We will only perturb $H_2(s,z)$ in this example since $H_1(s)$ is zero everywhere. We use the same data as in Sect. 6.2, to which we add uniformly distributed noise.

We apply AAA-LQO with $n_{\mathrm{max}}=40$ and $\tau = 10^{-2}$ for various noise levels, and thus obtain LQO models of various orders *n* as depicted in Table 2. For this experiment, increasing the noise level also increases the order of the fitted LQO system by means of AAA-LQO, which is to be expected. It is to be noted that for the higher level of noise considered here, namely $\zeta = 3 \cdot 10^{-3} M_2$ and $\zeta = 4 \cdot 10^{-3} M_2$, the target tolerance value $\tau =10^{-2}$ is not reached.

**Table 2:** Noise level $\zeta$ versus the order *n*

$$\zeta $$	0	$$ 2 \cdot 10^{-4} M_2$$	$$ 5 \cdot 10^{-4} M_2$$	$$ 10^{-3} M_2$$	$$ 3 \cdot 10^{-3} M_2$$	$$ 4 \cdot 10^{-3} M_2$$
*n*	24	24	30	34	40	40

**Fig. 10:**
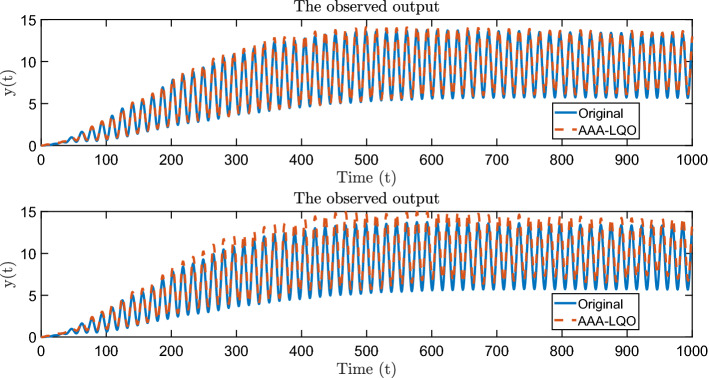
Time-domain simulations for the noisy data case for $\zeta = 3 \cdot 10^{-3} M_2$ (top plot) and $\zeta = 4 \cdot 10^{-3} M_2$ (bottom plot)

In the top plot in Fig. 10, we show the time-domain response of the full model and AAA-LQO model for the noise level of $\zeta = 3 \cdot 10^{-3}$, illustrating that the data-driven model accurately recovers the original model response. We repeat the same experiment with the noise level $\zeta = 4 \cdot 10^{-3}$ and depict the result in the bottom plot in Fig. 10, illustrating that the data-driven model starts to visibly deviate from the true model response. As expected, the approximation quality decays as the noise level increases.

Even though in this simple experiment AAA-LQO performs well for low to moderate noise levels, a more in-depth theoretical analysis on the robustness of AAA-LQO to noisy data together with algorithmic considerations (stopping criterion based on noise level, regularization etc.) is necessary and will be considered in future work.

## Conclusions

We have proposed a novel data-driven modeling method, called AAA-LQO, for linear systems with quadratic outputs (LQO). AAA-LQO extends the AAA algorithm to this new setting by first developing the barycentric representation theory for the two transfer functions arising in the analysis of LQO systems and then formulating a LS minimization framework to efficiently solve for the barycentric coefficients. The two numerical examples illustrate that AAA-LQO provides high-fidelity data-driven approximants to the original model.

The barycentric form we developed here for LQO systems offers promising research directions for modelling systems with general polynomial observation maps, as well as for nonlinearities appearing in the dynamical equation such as bilinear or quadratic-bilinear systems. These topics are the focus of on-going research.

## Data Availability

Enquiries about data availability should be directed to the authors.

## Proof of Lemma 4.1

Substitute $s = {\xi }_i$ and $z = {\widehat{{\xi }}}_j$ into (4.19) to obtain

$$\begin{aligned} \mathfrak {p}_2({\xi }_i,{\widehat{{\xi }}}_j) = \displaystyle \sum _{k=1}^n \sum _{\ell =1}^n h_{k,\ell }w_k w_\ell \mathfrak {M}_{k,\ell }({\xi }_i,{\widehat{{\xi }}}_j) = \displaystyle \sum _{\ell =1}^n h_{i,\ell }w_i w_\ell \mathfrak {M}_{i,\ell }({\xi }_i,{\widehat{{\xi }}}_j), \end{aligned}$$

and

$$\begin{aligned} \mathfrak {q}_2({\xi }_i,{\widehat{{\xi }}}_j)&= \mathfrak {M}({\xi }_i,{\widehat{{\xi }}}_j)+\displaystyle \sum _{k=1}^n w_k \mathfrak {m}_k({\xi }_i) \mathfrak {m}({\widehat{{\xi }}}_j) + \displaystyle \sum _{\ell =1}^n w_\ell \mathfrak {m}_\ell ({\widehat{{\xi }}}_j) \mathfrak {m}({\xi }_i)\\&\quad +\sum _{k=1}^n \sum _{\ell =1}^n w_k w_\ell \mathfrak {M}_{k,\ell }({\xi }_i,{\widehat{{\xi }}}_j) \\&= w_i \mathfrak {m}_i({\xi }_i) \mathfrak {m}({\widehat{{\xi }}}_j)+\sum _{\ell =1}^n w_i w_\ell \mathfrak {M}_{i,\ell }({\xi }_i,{\widehat{{\xi }}}_j), \end{aligned}$$

where $\mathfrak {M},\mathfrak {m},\mathfrak {m}_k$, and $\mathfrak {M}_{k,l}$ are as defined in (4.17). Write $r_2({\xi }_i,{\widehat{{\xi }}}_j) = \frac{\mathfrak {p}_2({\xi }_i,{\widehat{{\xi }}}_j)}{\mathfrak {q}_2({\xi }_i,{\widehat{{\xi }}}_j)}$ as

A.1 $$\begin{aligned} \begin{aligned} r_2({\xi }_i,{\widehat{{\xi }}}_j)&= \frac{\displaystyle \sum _{\ell =1}^n h_{i,\ell }w_i w_\ell \mathfrak {M}_{i,\ell }({\xi }_i,{\widehat{{\xi }}}_j)}{w_i \mathfrak {m}_i({\xi }_i) \mathfrak {m}({\widehat{{\xi }}}_j)+\displaystyle \sum _{\ell =1}^n w_i w_\ell \mathfrak {M}_{i,\ell }({\xi }_i,{\widehat{{\xi }}}_j)}. \end{aligned} \end{aligned}$$

Introduce the notation

A.2 $$\begin{aligned} \mathfrak {M}^L_{i}({\xi }_i,z) = \prod _{k=1, k \ne i}^n \prod _{\ell =1}^n (\xi _i-\xi _k) (z-\xi _\ell ) = \mathfrak {m}_i({\xi }_i) \mathfrak {m}(z). \end{aligned}$$

Since $\mathfrak {M}^L_{i}({\xi }_i,{\widehat{{\xi }}}_j) = \mathfrak {M}_{i,\ell }({\xi }_i,{\widehat{{\xi }}}_j) ({\widehat{{\xi }}}_j-{\xi }_\ell )$ holds, we can write

A.3 $$\begin{aligned} \begin{aligned} r_2({\xi }_i,{\widehat{{\xi }}}_j)&= \frac{\displaystyle \sum _{\ell =1}^n h_{i,\ell }w_i w_\ell \frac{\mathfrak {M}^L_{i}({\xi }_i,{\widehat{{\xi }}}_j)}{{\widehat{{\xi }}}_j-{\xi }_\ell }}{w_i \mathfrak {M}^L_{i}({\xi }_i,{\widehat{{\xi }}}_j)+\displaystyle \sum _{\ell =1}^n w_i w_\ell \frac{\mathfrak {M}^L_{i}({\xi }_i,{\widehat{{\xi }}}_j)}{{\widehat{{\xi }}}_j-{\xi }_\ell }}. \end{aligned} \end{aligned}$$

By simplifying $w_i \mathfrak {M}^L_{i}({\xi }_i,{\widehat{{\xi }}}_j)$ from both the numerator and the denominator in the above expression proves the first desired result in (4.23). The proof for $r_2({\widehat{{\xi }}}_j,{\xi }_i)$ follows similarly.

## Footnotes

1 With the addition of 1 to the denominator, we guarantee that *r*(*s*) is a strictly proper rational function with a numerator degree $n-1$ and the denominator degree *n*. This is done in the anticipation of the dynamical system in (2.1) we aim to approximate where there will be no direct input-to-output mapping. This is not a restriction, and the numerator and denominator degrees can be chosen in a different way [11, 32].

2 Since the evaluation of the uninterpolated $H_2(s,z)$ values occur over three different sets, namely ${\varvec{{\xi }}}\times {\widehat{{\varvec{{\xi }}}}}$, ${\widehat{{\varvec{{\xi }}}}}\times {\varvec{{\xi }}}$, and ${\widehat{{\varvec{{\xi }}}}}\times {\widehat{{\varvec{{\xi }}}}}$, we use a superscript to distinguish them. Recall that the interpolated values $h_{i,j} = H_2({\xi }_i,{\xi }_j)$ are over ${\varvec{{\xi }}}\times {\varvec{{\xi }}}$ only and thus the superscript notation is avoided for $h_{i,j}$.
